# The impact of Centre’s heart transplant status and volume on in-hospital outcomes following extracorporeal membrane oxygenation for refractory post-cardiotomy cardiogenic shock: a meta-analysis

**DOI:** 10.1186/s12872-019-01317-y

**Published:** 2020-01-09

**Authors:** Mariusz Kowalewski, Giuseppe Maria Raffa, Kamil Zieliński, Musab Alanazi, Martijn Gilbers, Sam Heuts, Ehsan Natour, Elham Bidar, Rick Schreurs, Thijs Delnoij, Rob Driessen, Jan-Willem Sels, Marcel van de Poll, Paul Roekaerts, Paolo Meani, Jos Maessen, Piotr Suwalski, Roberto Lorusso

**Affiliations:** 1 Department of Cardiac Surgery, Central Clinical Hospital of the Ministry of Interior, Centre of Postgraduate Medical Education, Warsaw, Poland; 2 Cardio-Thoracic Surgery Department, Maastricht University Medical Centre, Cardiovascular Research Institute Maastricht (CARIM) , Maastricht, Netherlands; 3 Cardiothoracic Research Centre, Innovative Medical Forum, Bydgoszcz, Poland; 4 Department for the Treatment and Study of Cardiothoracic Diseases and Cardiothoracic Transplantation, ISMETT-IRCCS, Palermo, Italy; 5 Medical University of Warsaw, Warsaw, Poland; 6 Cardiology Department, Maastricht University Medical Centre, Maastricht, Netherlands; 7 Department of Intensive Care, Maastricht University Medical Centre, Maastricht, Netherlands

**Keywords:** Extracorporeal membrane oxygenation, Extracorporeal life support, Cardiogenic shock, Meta-analysis

## Abstract

**Background:**

Postcardiotomy cardiogenic shock (PCS) that is refractory to inotropic support remains a major concern in cardiac surgery and is almost universally fatal unless treated with mechanical support. While reported mortality rates on ECMO vary from center to center, aim of the current report is assess if the outcomes differ between centres according to volume and heart transplantation status.

**Methods:**

A systematic search was performed according to PRISMA statement using PubMed/Medline databases between 2010 and 2018. Relevant articles were scrutinized and included in the meta-analysis only if reporting in-hospital/30-day mortality and heart transplantation status of the centre. Paediatric and congenital heart surgery-related studies along with those conducted in the setting of veno-venous ECMO for respiratory distress syndrome were excluded. Differences were assessed by means of subgroup meta-analysis and meta-regression.

**Results:**

Fifty-four studies enrolling *N* = 4421 ECMO patients were included. Of those, 6 series were performed in non-HTx centres (204 pts.;4.6%). Overall 30-day survival (95% Confidence Intervals) was 35.3% (32.5–38.2%) and did not statistically differ between non-HTx: 33.3% (26.8–40.4%) and HTx centres: 35.7% (32.7–38.8%); P_interaction_ = 0.531. There was no impact of centre volume on survival as well: ß_coef_ = 0.0006; *P* = 0.833. No statistical differences were seen between HTx and non-HTx with respect to ECMO duration, limb complications, reoperations for bleeding, kidney injury and sepsis. There were however significantly less neurological complications in the HTx as compared to non-HTx centres: 11.9% vs 19.5% respectively; *P* = 0.009; an inverse relationship was seen for neurologic complications in centres performing more ECMOs annually ß_coef_ = − 0.0066; *P* = 0.031. Weaning rates and bridging to HTx and/or VADs were higher in HTx facilities.

**Conclusions:**

There was no apparent difference in survival after ECMO implantation for refractory PCS according to centre’s ECMO volume and transplantation status. Potentially different risk profiles of patients in these centres must be taken account for before definite conclusions are drawn.

## Background

Extracorporeal Membrane Oxygenation (ECMO) use is increasing; yet, it still does represent a resource-consuming modality of treatment and, in majority of cases, is seen as a last resort for patients who, otherwise, would inevitably die [1–5]. Postcardiotomy- and ST-elevation myocardial infarction (MI) complicating- cardiogenic shock (CS) were two most frequent indications for VA-ECMO implantation in the United States until 2011 [3–5]. Despite growing worldwide utilization and experience in mechanical circulatory support (MCS), in particular, in-hospital outcomes while on ECMO have not shown substantial progress [6]. While little is still known on who benefits most from ECMO support which is a long and advanced therapy [7], European Society of Cardiology guidelines cautiously assigned ECMO class of recommendation IIb, level of evidence C for the management of cardiogenic shock in STEMI [8].

Unlike STEMIs, cardiac surgical patients are usually characterized by substantial pre-ECMO comorbidities and more advanced age [9]. All these factors, individually or in association, may inhibit the potential of myocardium to recover after the surgery and/or hamper favorable body response to prolonged MCS. Indeed, in some patients, prolonged MCS does not lead to improved cardiac function or organ integrity; clinicians are therefore forced to bridge the patient; since bridge to recovery is no longer an option, more advanced treatments, such as heart transplantation (HTx) or long-lasting ventricular assist devices (VADs) remain. Not all heart surgery centres perform HTx, and not all of them perform VADs.

We therefore, undertook systematic review and meta-analysis to assess to which extent do the in-hospital outcomes differ across PCS-ECMO recipients in heart transplantation- as compared to non-transplant units to which could be attributed to readability of ECMO teams and potentially shorter bridging times in HTx/VAD units. To assess the impact of possible differences in experience between centres, we analyzed how the in-hospital outcomes are affected by centre’s volume and annual ECMO institution rates.

## Methods

### Data sources and search strategy

This systematic review and meta-analysis was performed in accordance with the Preferred Reporting Items for Systematic Reviews and meta-analyses (PRISMA) statement [10]. The PRISMA checklist is available as Appendix. To best reflect current clinical practice, relevant studies to be included were searched for between year 2000 until March 31st 2018, through PubMed, EMBASE, CINAHL, the Web of Science, the Cochrane Register of Controlled Clinical Trials (CENTRAL) and Google Scholar. Abstracts were eligible for detailed assessment if available online and reporting outcomes of interest. The search term was: “extracorporeal membrane oxygenation” and “extracorporeal life support”. No language restrictions were imposed. References of original articles were reviewed manually and cross-checked for other relevant reports.

### Selection criteria and quality assessment

Studies were included if they met all of the following criteria: 1) human study; 2) studies assessing survival after ECMO instituted for postcardiotomy refractory cardiogenic shock; 3) study reporting institutional outcomes that for ECMO indication combined postcardiotomy and non-postcardiotomy cardiogenic shock but reporting outcomes of interest separately for the groups. Studies were excluded if: 1) paediatric and congenital heart surgery-related studies; 2) animal studies; 3) conducted in the setting of veno-venous ECMO for respiratory distress syndrome; and 4) studies not reporting survival/mortality rates. Studies were only eligible if reporting the transplant status of the centre; whenever this was not retrievable from the individual study, institutional website was searched for information regarding range of procedures performed. Lack of clear indication whether the centre performs heart transplantation led to exclusion of the study. Similarly, registries incorporating multiple centres but not reporting the status for single facilities were not considered. Reviews and case reports were not considered.

Two independent reviewers (P.M. and K.Z.) selected the studies for inclusion, extracted studies, as well as patient characteristics of interest and relevant outcomes. Two authors (P.M. and K.Z.) independently assessed the trials’ eligibility and risk of bias. Risk of bias at the individual study level was assessed using the ROBINS-I tool (Risk of Bias in Not-randomized Studies-of Interventions) [11]. Any divergences were resolved by a third reviewer (R.L.) and quantified using the approach of Cohen’s kappa [12].

### Endpoint selection

The primary endpoint was in-hospital survival. Secondary endpoints were in-hospital cerebrovascular events (CVE), limb complications, bleeding or reoperation for bleeding, sepsis and acute kidney failure with or without continuous veno-venous hemofiltration (CVVH). Bridging to VAD and/or HTx was analysed as well. Outcome definitions were the ones adopted by the investigators of the included studies.

### Statistical analysis

Statistical analyses were performed in Comprehensive Meta-Analysis, v. 2 (Biostat,

Englewood, NJ). The results are expressed as pooled untransformed proportions (eg. event rates (%) and means with their 95% confidence intervals (CI). Heterogeneity across studies was evaluated using the *I*^2^ test. Where available, we digitised Kaplan-Meier curves using Engauge Digitizer 9.5 (Mark Mitchell, Torrance, CA) and reconstructed time-to-event data using the algorithm specified by Guyot et al. [13]. To control for the anticipated heterogeneity among observational studies, absolute values and means were pooled using random effects models. Studies were stratified a priori based on the centre status (HTx vs non-HTx performing centre); the interaction coefficient (Q-value) is provided for the comparison HTx vs non-HTx along with respective P_interaction_. Additionally, we investigated if HTx and non-HTx status had influence on ECMO duration, weaning rates, bridging to HTx/VAD rates; and further if ECMO duration and weaning rates in these centres correlated with bridging to HTx/VAD by means of meta-regression analyses [14]. Similarly meta-regression approach was used to determine whether annual ECMO institution rate for centre reporting such, affects the survival and remaining in-hospital outcomes. Annual ECMO institution rate was calculated by dividing number of study subjects by study duration period. Sensitivity analyses were performed by excluding from analyses single studies, one at a time, and repeating the calculations. Subgroup analyses were performed for survival endpoint by dividing the studies into distinctive strata (by mean and median annual ECMO institution rate as well as in tertiles and quartiles) and reporting respective P_interaction_ for between subgroup comparison. Publication bias was assessed 1) by visual approach plotting log event rate against standard error in the funnel plot; and 2) by linear regression approach [15].

## Results

Initial search process yielded 22,609 records; of these, 183 abstracts were retrieved for scrutiny based on the item’s title. Registries were excluded since they incorporated both HTx and non-HTx centres [16–18]. Following detailed assessment, 54 studies (*N* = 4421 patients) [list of references to included studies] met inclusion criteria and entered quantitative analyses. PRISMA flow chart is available as Additional file 1: Figure S7. Included studies were divided into HTx vs non-HTx centres subgroups: 48 studies including 4217 (95.4%) patients were conducted in HTx- whereas 6 studies (*N* = 204) in non-HTx centres. Prevalence of ECMO ranged from 0.26% [70] to 3.35% [28]. Patients receiving ECMO at HTx centres were significantly younger than their non-HTx counterparts 57.2 ± 1.6 vs 64.2 ± 1.6 *P* < 0.001. CABG was most frequent procedure in both HTx and non-HTx centres 33.7 and 30.9% followed by valvular (25.1 and 21.1%) and combined surgeries (16.5 and 26.5%). Detailed characteristics of included studies as well as patients’ baseline and surgical data are available in Table 1. Publication bias analysis along with reasons for bias risk increase is available as Additional file 1: Table S1; studies were judged to be moderate to severe risk of bias as none previously compared directly HTx vs non-HTx centre performance; no signs of asymmetry were seen on visual inspection of funnel plot for primary endpoint (Additional file 1: Figure S8).

**Table 1:** Study characteristics

Study	Study time frames	Prevalence (%)	N. of pts	Age (years)	Male(%)	LVEF (%)	Baseline sCr (mg/dL)	Status elective/ urgent/ emergency/ salvage (n)	Surgery (n)					
									CABG	Valve	Combined	Other	CPB	X-clamp
HTx/VAD centres														
Acheampong 2016	2001–2013	1.06	24	41 (IQR: 22–75)	58.3	47 (10–66)	NR	NR	0	14	3	7	NR	NR
Bakhtiary 2008	2003–2006	0.78	45	60.1 ± 13.6	77.8	25.8 ± 10	NR	NR	20	2	11	12	NR	NR
Beckmann 2017	1997–2011	NR	8	50.1 ± 15.8	62.5	NR	NR	NR/NR/3/NR	0	4	1	3	NR	NR
Beiras-Fernandez 2011	1996–2006	NR	73	49.3 ± 18.0	64.4	NR	1.8 ± 1.1	NR	14	13	6	40	254.3	110.9
Biancari 2017	2005–2016	0.60	148	65.4 ± 9.4	78.4	NR	NR	19/34/80/15	148	0	0	0	146	68
Burrell 2015	2007–2013	NR	54	48 (IQR: 34–58)	68.5	NR	1.2 (0.9–1.6)	NR	NR				NR	NR
Carroll 2015	2009–2014	NR	26	56 (IQR: 41–65)*	69.1*	30 (17–57)*	NR	NR	NR				NR	NR
Chen 2011*	2002–2008	NR	60	47 ± 2	60.8	NR	1.3 ± 0.1	NR	NR				NR	NR
Combes 2008	2003–2006	NR	16	46 ± 16*	56.8*	NR	NR	NR	NR				NR	NR
Distelmeier 2016	2003–2014	3.35	354	65 (IQR: 55–73)	68.6	NR	1.3 (1.1–1.8)	NR	48	110	84	111	NR	NR
Doll 2003	1997–2000	1.20	95	59.8 ± 13.3	69.5	46 ± 17.4	NR	21/64/10/0	63	18	8	6	NR	NR
Elsharkawy 2010	1995–2005	0.58	233	NR	67.4	NR	NR	NR/NR/84/NR	NR				NR	NR
Fiser 2001	1993–2000	0.91	51	61.0 ± 1.7	56.9	NR	NR	NR	34	5	5	7	172.3	68.6
Guihaire 2017	2005–2014	0.70	92	64.5 (18–83)	59	47.2 ± 17.4	1.16 ± 0.58	NR/NR/33/NR	12	64	31**	16	173.3	109.5
Hsu 2009	2002–2006	2.89	51	63.0 ± 15.7	70.6	40.1 ± 17.9	NR	NR	27	11	7	6	188	32
Kanji 2010*	2002–2006	NR	50	49.36	72	23.0	NR	NR	NR				NR	NR
Ko 2002	1994–2000	2.61	76	56.8 ± 15.9	63.2	NR	NR	NR	37	14	6	19	NR	NR
Lamarche 2010	2000–2008	0.30	24	54.6 ± 21	63	20 ± 25	NR	NR	NR				NR	NR
Li 2015	2011–2012	0.91	123	56.2 ± 11.8	65.9	54.6 ± 13.6	NR	NR	44	40	15	24	NR	NR
Liden 2009	2000–2007	NR	33	52.4 ± 12.7	93.9	NR	NR	NR	10	0	0	23	NR	NR
Liu 2009	2002–2005	0.58	14	55.7 ± 15.4	50	52.9 ± 21.4	1.3 ± 1.1	NR	1	7	2	4	247	132
Loforte 2014	2006–2012	1.27	155	58.2 (23–84)*	65.8*	48.2 (33–75)*	NR	NR	28	43	32	52	214.5	NR
Luo 2009	2005–2008	NR	36	49.7	72.2	NR	NR	NR	15	8	0	13	NR	NR
Mazzeffi 2016	2010–2015	0.42	23	57 ± 15	60.9	50 (40–55)	1.2 (0.9–2.0)	NR	7	6	0	10	160	NR
Meyer 2009	2007–2007	1.23	18	50 ± 15	72.2	NR	NR	NR	5	2	0	10	237	NR
Musial 2017	2009–2016	NR	27	45 ± 16	70.4	33.1 ± 22.3	NR	NR	0	16	0	11	NR	NR
Papadopoulos 2015	2001–2013	NR	360	62 ± 17	76.1	35	NR	NR/NR/NR/50	114	89	85	72	160	71
Park 2014	2005–2012	NR	115	61.7 ± 13.4	48.7	57.1 ± 14.3	NR	NR	25	31	33	26	232.4	104.7
Peigh 2015	2010–2014	NR	13	48 ± 14*	64.4*	NR	1.5 ± 0.8*	NR	NR				NR	NR
Pokersnik 2012	2005–2010	NR	49	65 ± 13	67.3	NR	NR	NR/NR/0/0	NR				239	110
Pontailler 2017	2004–2014	NR	127	75.7 ± 4.7*	61.3*	23 ± 12	1.9 ± 1.0	NR/NR/49/NR	42	36	18	18	NR	NR
Ranucci 2011	2008–2011	NR	11	NR	NR	NR	NR	NR			NR		283.1	NR
Rastan 2010	1996–2008	1.28	517	63.5 ± 11.2	71.5	19.9	NR	159/122/205/31	193	96	72	156	179	84.6
Rousse 2015	NR	NR	29	50.6 ± 15.4	65.5	NR	NR	NR	NR				NR	NR
Rubino 2017	2008–2016	NR	101	57.1 ± 15.8	63.4	NR	NR	NR	0	12	29	60	278.2	NR
Russo 2010	2005–2009	NR	3	63.3 ± 14.5	66.7	NR	1,3 ± 0.1	NR	1	1	0	1	NR	NR
Saxena 2015^†^	2003–2013	NR	45	76.8 ± 4.6	68.9	48.5	1.4 ± 0.5	35/6/4/0	NR				NR	NR
Slottosch 2012	2006–2010	NR	77	60 ± 13	76.6	42 ± 19	NR	NR/NR/29/NR	43	10	11	13	173	69
Slottosch 2017	2008–2016	NR	100	58 ± 15*	76.3	NR	NR	NR/NR/37/0	45	19	24	12	170	68
Truby 2015 *	2007–2013	NR	70	56.9 ± 16.1	67.6	NR	1.83 ± 1.09	NR	11	21	NR	8	NR	NR
Tsai 2016	2002–2011	NR	67	57 ± 14*	66.7*	NR	1.71 ± 2.17*	NR			NR		NR	NR
Wang 2009	2004–2008	0.49	62	51 ± 15	51.6	50 ± 16	NR	NR	13	39	4	6	NR	NR
Wang 2013	2004–2011	1.79	87	65 ± 7	58.6	46 ± 12	NR	NR			NR		182	94
Wu 2010	2003–2009	2.63	110	60 ± 14	70.9	43.4 ± 19.2	NR	NR/NR/31/NR	31	42	19	18	224	126
Xie 2017***	2011–2015	NR	273	NR	NR	NR	NR	NR			NR		NR	NR
Zhang 2006	1996–2004	NR	32	55.4 ± 11.9	56.3	55.3 ± 14.8	NR	NR	5	10	12	5	237	98.7
Zhao 2015	2004–2012	NR	24	59.3 ± 11.9	79.2	51.8 ± 14.2	NR	NR	20	2	0	2	NR	NR
Zhong 2017	2009–2016	0.64	36	50.4 ± 12.2	91.7	60.6 ± 7.86	NR	NR/NR/9/NR			NR		279	113
Non-HTx/VAD centres														
Ariyaratnam 2014	2003–2013	NR	14	65.6 ± 10.5	57.1	NR	NR	NR	0	0	11	3	205.6	109.6
Deschka 2013	2008–2011	NR	28	66.6 ± 5.3	92.9	37.2 ± 13.0	NR	NR	17	11	0	0	NR	NR
Khorsandi 2016	1995–2015	NR	15	64.3 ± 14.5	73.3	NR	NR	9/3/4/0	3	4	5	3	NR	NR
Mikus 2013	2007–2011	0.26	14	53.1 ± 14.3	64.3	46.1 ± 13.9	NR	6/2/6/0	5	6	3	0	240.5	98.4
Raffa 2017	2007–2017	NR	86	65 ± 11.2	65.1	NR	NR	NR/NR/33/NR	19	14	29	24	197	104
Unosawa 2012	1992–2007	NR	47	64.4 ± 12.5	74.4	47.8 ± 19.1	NR	NR/NR/22/NR	19	8	6	14	217	81.2

* Reported for entire study population including non-PCS patients

** Multiple valve repair or valvular surgery combined with CABG; potential overlap

*** Article in Chinese – data from English abstract

^†^One patient had transfemoral transcatheter aortic valve replacement

Values are reported as mean ± SD, unless reported otherwise in the original manuscript. Numbers of patients in single studies do not always match the total n. of patients in the original manuscripts since only PCS subgroups were considered. Slottosch 2012 and Slottosch 2017 are studies from the same group; first [Additional file 1 reference 39] describes ECMO support after surgery for acquired heart disease from 2006 to 2010, the second [Additional file 1: reference 40] reports all patients receiving ECMO support longer than 48 h for cardiogenic shock from 01/2008 to 12/2016 therefore, there exists a potential overlap of included patients. LVEF, left ventricle ejection fraction; sCr, serum creatinine; CABG, coronary artery bypass grafting; CPB, cardiopulmonary bypass; IQR, interquartile range; NR, not reported

### ECMO strategy

In the studies that reported procedural details, ECMO was established during the initial cardiac surgery in 42.7% of cases because of circulatory instability during or immediately after weaning from cardiopulmonary bypass. ECMO was initiated in the OR in 56.5% of patients (50.1–62.7%), followed by ICU, cardiac catheterization laboratory, telemetry floor and emergency department. There was no significant difference in the rate of placement of ECMO in the OR in non-HTx - as compared to HTx centres with respective rates of 64.5% (52.9–74.6%) vs 53.2% (45.6–60.7%); *P* = 0.108. Peripheral cannulation was preferred approach (69.0%) for ECMO institution. Median ECMO duration in the entire series was 5 days (IQR: 3.3–6.0); without apparent differences between HTx (mean weighted average = 4.92 days) vs non-HTx- (5.04 days) centres. The details of procedural characteristics are available as Additional file 1: Table S2. Successful weaning from ECMO was most often defined as decannulation after > 48 h. Overall, estimated 55.3% patients were weaned from ECMO with the weaning rates ranging from 31.4–100% in the entire series. No difference was noted regarding weaning rates between HTx vs non-HTx centres (56.6% vs 50.4%; *P* = 0.118).

### Survival and complications while on ECMO

**Fig. 1:**
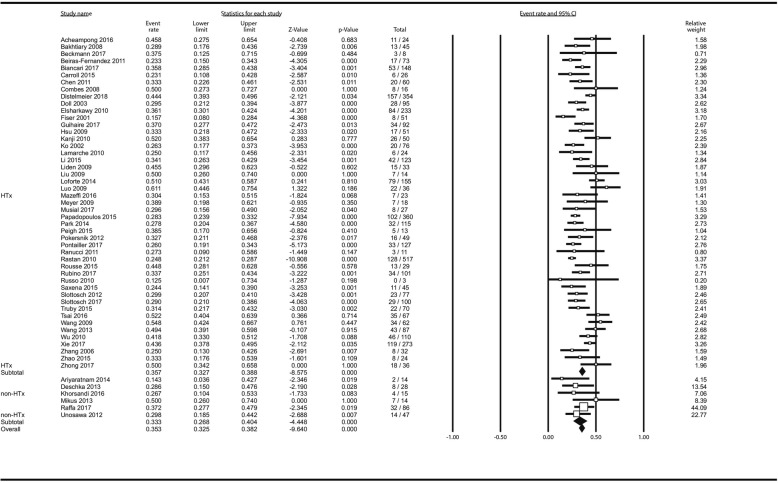
Analysis of survival after following ECMO institution in HTx/VAD vs non-HTx/VAD centres. Squares represent point estimates of single studies; horizontal lines are respective 95% confidence intervals. Diamonds are indicative of subtotal and total pooled estimate. HTx, heart transplantation; VAD, ventricle assist device

Reported causes of death were divided into “while on-ECMO” and “after weaning” and are available in Additional file 1: Table S3. Fifty-three studies (4367 patients) contributed to the analysis of survival: Overall, 1527 patients survived to hospital discharge which translated to estimated overall survival of 35.3% (32.5–38.2%). There was no difference between HTx - (35.7% [32.7–38.8%]) and non-HTx centres (33.3% [26.8–40.4%]) *p* = 0.531 in random effects model. Figure 1. In meta-regression, there was no impact of centre volume on survival as well: ß_coef_ = 0.0006; *P* = 0.833 (Additional file 1: Figure S9).

**Fig. 2:**
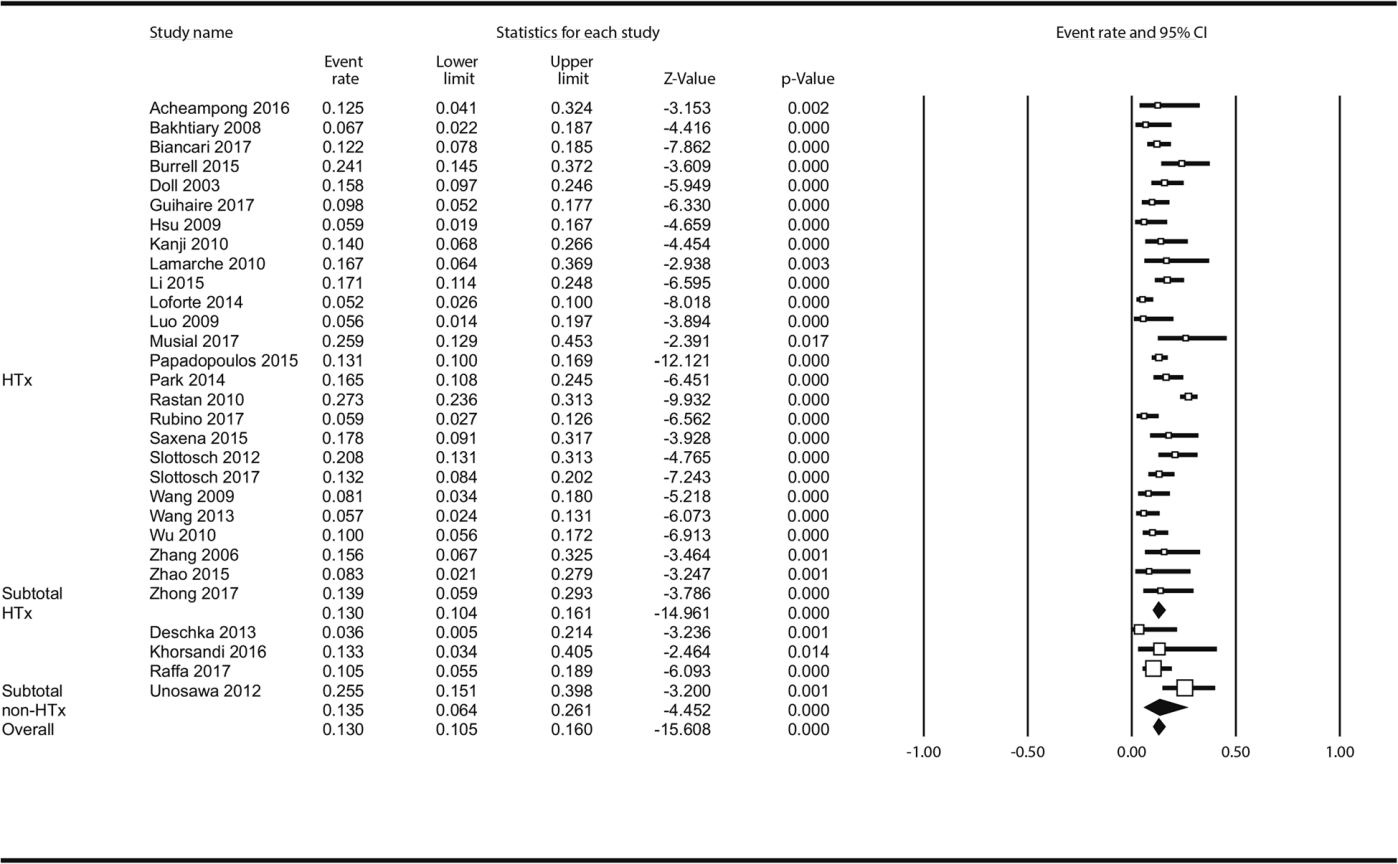
Analysis of limb complications following ECMO institution in HTx/VAD vs non-HTx/VAD centres. Abbreviations as in Fig. 1

Limb complications incidence was reported in 30 studies (2766 pts). Overall, 424 patients (13.0% [10.5–16.0%]) had limb complications; Fig. 2; in the analysis stratified by centre status there was no difference between HTx - (13.0% [10.4–16.1%] and non-HTx centres (13.35 [6.4–26.1%] *P* = 0.919). In meta-regression, there was no impact of centre volume on incidence of limb complications: ß_coef_ = 0.0043; *P* = 0.342 (Additional file 1: Figure S10).

**Fig. 3:**
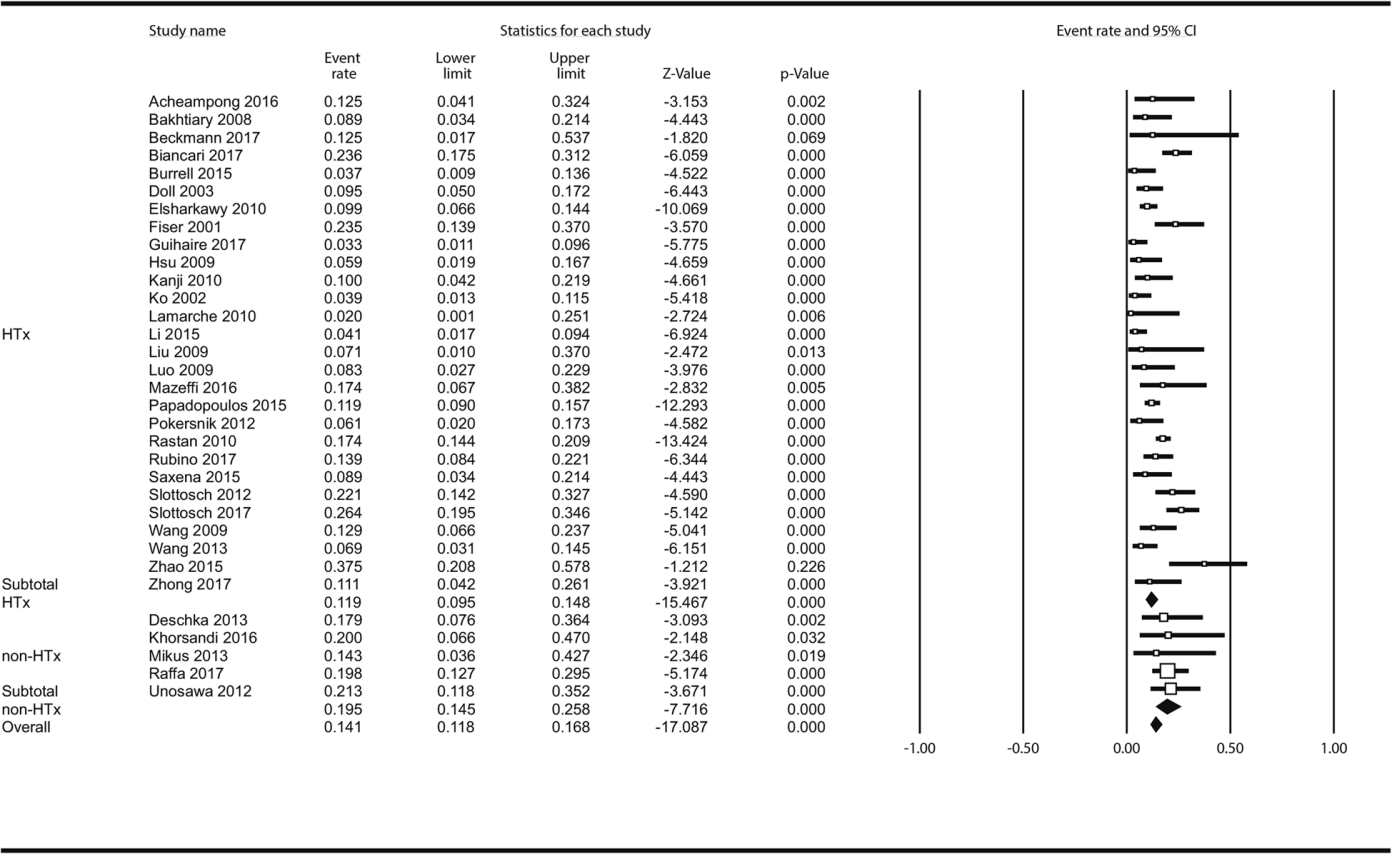
Analysis of neurologic complications following ECMO institution in HTx/VAD vs non-HTx/VAD centres. Abbreviations as in Fig. 1

There were significantly less neurological complications in the HTx as compared to non-HTx centres: overall 385 patients (33 studies) experienced neurological complications (14.1% [11.8–16.8%]) Fig. 3; among those 88 brain deaths (7.9% [5.6–11.0%] occurred. Additional file 1: Figure S11. Neurologic complications in non-HTx centres followed in 19.5% (14.5–25.8%) as compared to 11.9% (9.5–14.8%) in HTx centres; *P* = 0.009. In meta-regression, less neurologic complications and brain deaths were seen in centres with higher annual ECMO institution rate: ß_coef_ = − 0.0066; *P* = 0.031 (Additional file 1: Figure S12) and ß_coef_ = − 0.0515; *P* = 0.071 (Additional file 1: Figure S13) respectively for neurologic complications and brain deaths.

**Fig. 4:**
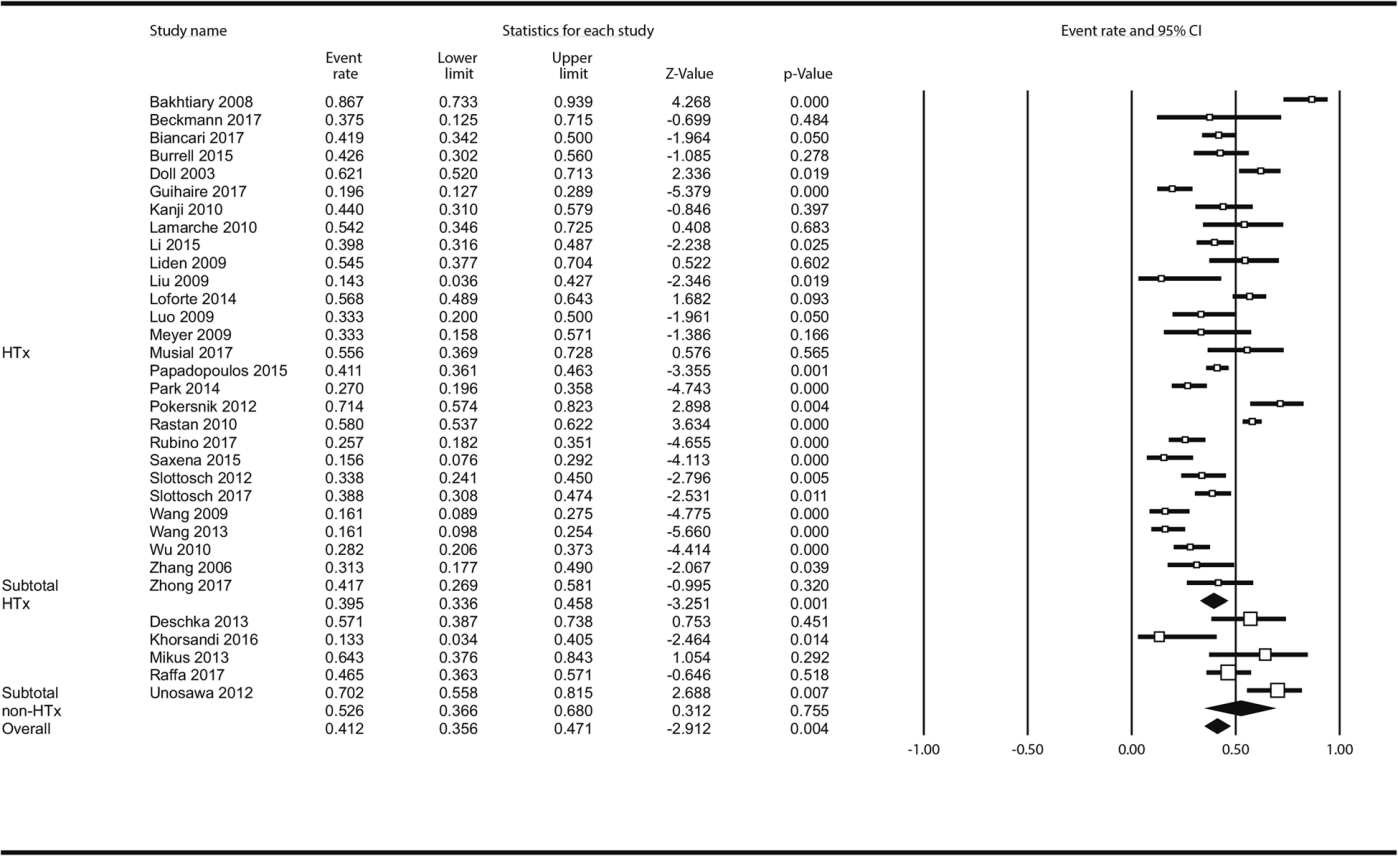
Analysis of reoperations for bleeding following ECMO institution in HTx/VAD vs non-HTx/VAD centres. Abbreviations as in Fig. 1

**Fig. 5:**
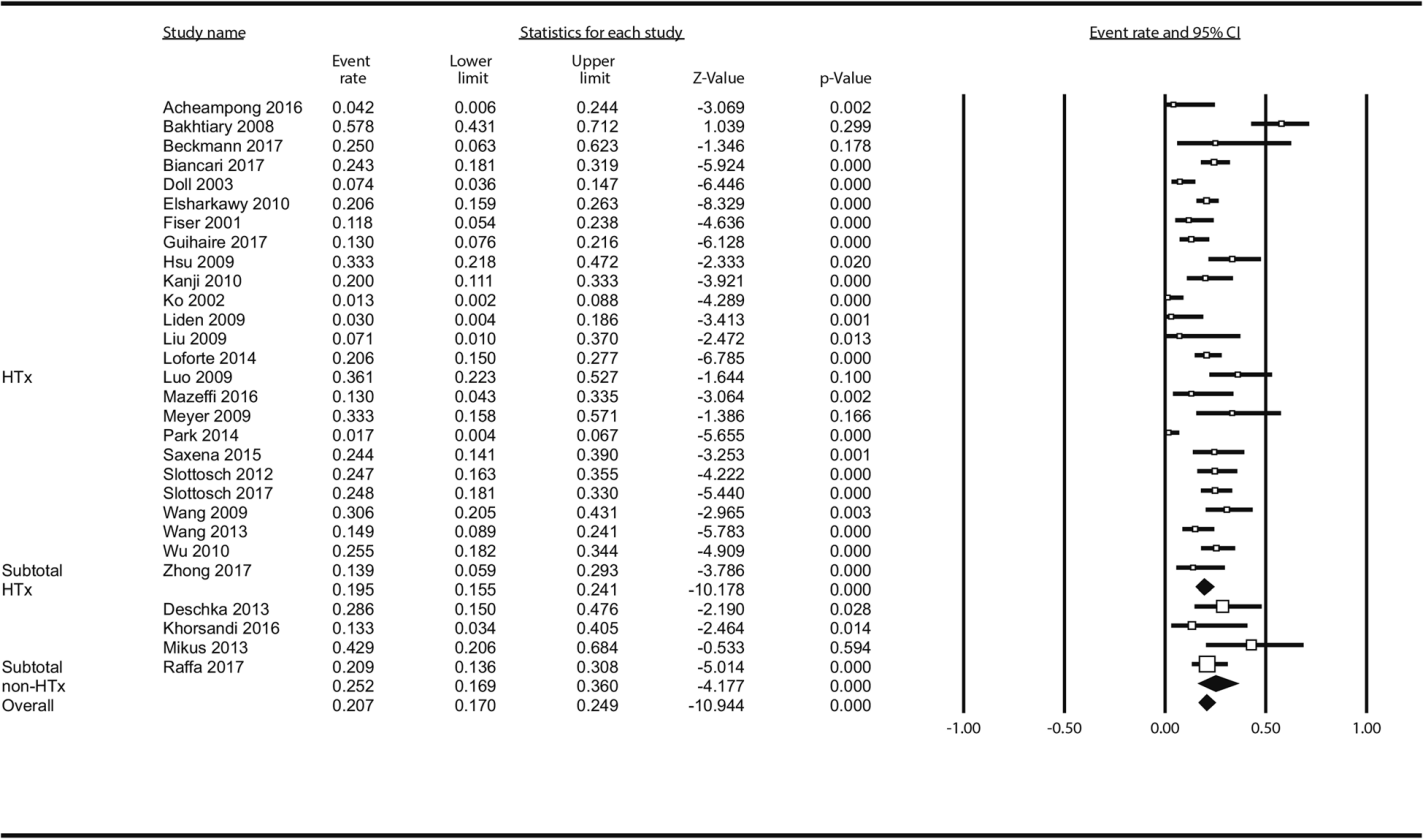
Analysis of sepsis following ECMO institution in HTx/VAD vs non-HTx/VAD centres. Abbreviations as in Fig. 1

**Fig. 6:**
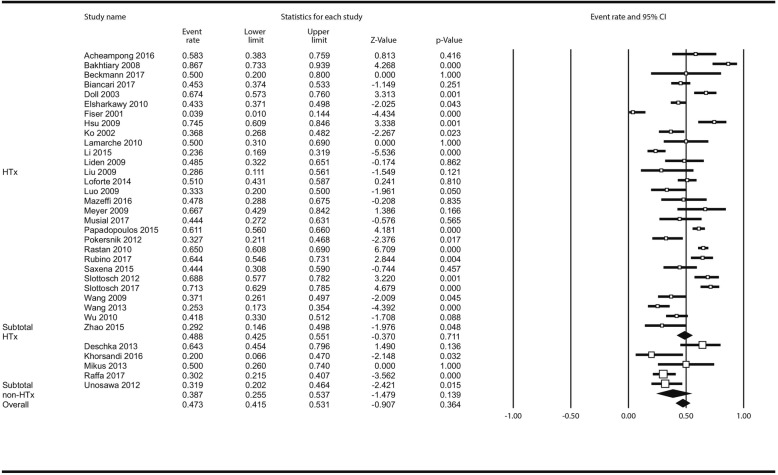
Analysis of acute kidney injury following ECMO institution in HTx/VAD vs non-HTx/VAD centres. Abbreviations as in Fig. 1

Thirty-three studies enrolling 2832 patients reported reoperations for bleeding; these were necessary in 1232 cases (41.2% [35.6–47.1%]) in the entire series without statistical differences between HTx: 39.5% (33.6–45.8%); and non-HTx centres: 52.6% (36.6–68.0%); *P* = 0.139. Figure 4. In meta-regression, there was no impact of centre volume on incidence of reoperation for bleeding: ß_coef_ = − 0.0012; *P* = 0.489 (Additional file 1: Figure S14). Sepsis has complicated 385 ECMO cases 20.7% (17.0–24.9%) but there were again no differences between HTx - (19.5% [15.5–24.1%]) and non-HTx centres (25.2% [16.9–36.0%]); *P* = 0.259 in the meta-analysis (Fig. 5) nor in meta-regression of centre’s volume impact (Additional file 1: Figure S15) (ß_coef_ = − 0.0040; *P* = 0.692). In the analysis of AKI with or without CVVH (Additional file 1: Table S4 lists AKI definitions across included studies) less AKIs in non-HTx centres were seen but the difference was not significant (*p* = 0.220) Fig. 6: Total incidence of AKI was 47.3% (41.5–53.1%) – 1513 reported cases; in non-HTx centres AKI estimated rate was 38.7% (25.5–53.7%) as compared to 48.8% (42.5–55.1%) as observed in HTx centres; no effect of centre’s annual ECMO institution rate on AKI incidence was demonstrated in meta-regression (ß_coef_ = − 0.0012; *P* = 0.488) Additional file 1: Figure S16.

### ECMO as bridging therapy

Eighty-six (estimated rate 3.5% [1.8–6.6%]) patients were bridged to heart transplantation. Of those, all were bridged to HTx in HTx centres. Off note, one reported patient died on ECMO after transfer from non-Htx centre to the referral hub centre while waiting for heart transplantation [70]. ECMO bridging to short- or long-term VAD ensued in 99 patients (4.3% [2.8–6.5%]); there were again no instance of reported bridging to VADs in non-HTx centres.

### Additional analyses

In several conducted meta-regressions, no impact of centre status on survival (ß_coef_ = 0.1418; P_slope_ = 0.555) or ECMO duration (ß_coef_ = 0.0052; P_slope_ = 0.833) could be demonstrated. Centre status positively, yet non-significantly, correlated with higher weaning rates (ß_coef_ = 0.2651; P_slope_ = 0.601). Additional file 1: Figure S17 and 12 summarize subgroup analyses performed for survival rates as divided by annual number of ECMOs performed. In sensitivity analysis for survival performed deleting single studies, one at a time, and repeating the calculations, no single study effect was seen changing neither direction nor the magnitude of the estimates.

## Discussion

The current meta-analysis represents the first attempt to compare, although in indirect fashion, in-hospital outcomes of patients supported with VA-ECMO for refractory PCS between HTx and non-HTx centres. This research was aimed to investigate further factors other than the well-known patients’ clinical status and procedure type that may affect the final outcome in PCS-ECMO patient. The care center with experience in dealing with acute and chronic end stage heart failure with expertise and prompt resources availability (medium and long term mechanical circulatory support and heart transplantation) as factor potentially affecting this outcome was the primary hypothesis of our study.

VA-ECMO is increasingly used for cardiorespiratory support in patients affected by refractory cardiogenic shock or cardiac arrest after cardiac surgery [2]. Despite that growing worldwide utilization and experience, ECMO in-hospital outcomes have not shown substantial progress. Conversely, a trend towards worse survival rates, reaching a disappointing 15% has been recently reported in another analysis of the Extracorporeal Life Support Organization registry [2]. Patients undergoing heart surgery usually present with substantial pre-ECMO comorbidities, more advanced age and above all different stages of developed heart failure. All these factors, individually or in association influence the capability of the myocardium to recover after the surgery and thus preclude favorable body response to prolonged MCS. Unfortunately, in a considerable proportion of patients, the MCS regardless of its duration, does not prompt to improved cardiac function or organ integrity; in turn, clinicians are forced to bridging the patient to more advanced treatments, such as HTx or VADs. The insights from important recently available study by Distelmeier [19] are that prolonging of VA-ECMO duration is associated with a disproportionate mortality at early and later stages. In fact, lack of cardiac function improvement within 7 days post-op. was indicative of futile support in the analysis. Consequently, this leads to conclusion that perhaps HTx or VADs in such ECMO-supported patients should be used much sooner, just in time to prevent life-threatening complications.

Such hypothesis led to conception of the current study which is the first to compare, although in indirect fashion, the outcomes between HTx and non-HTx performing centres in patients undergoing ECMO treatment for refractory PCS. The first consideration come from the study population of this meta-analysis: The majority of patients (4217 out of 4421) and number of reports (48 out of 54) come from HTx centres which might suggest that 1) cardiac surgery population in HTx performing centres are of an increased risk for developing PCS, therefore higher operative risk burden in a first place; 2) ECMO represents a tool more routinely used for the treatment of refractory PCS in the HTx units; 3) there exists an unexplained underreporting from non-HTx centres of patients undergoing ECMO treatment and their perioperative outcomes. Regardless, our main findings were that among patients with PCS no differences in 30 day/in-hospital mortality were observed between heart transplantation centres as compared to non-transplant units. This was also confirmed in a subgroup analysis. While neutral, this finding implies similar mortality rates among patients operated on in HTx and long-term assistance facilities as compared to patients operated in non-transplant units, given their respective potentially higher and lower baseline risk.

Second, there was no difference between the centres type with respect to limb complications, reoperation for bleeding, sepsis and acute renal injury with or without dialysis, yet neurological complications occurred less frequently in HTx centres. Neurologic complications are presumably a multifactorial entity with pre-ECMO illness severity and treatments, ECMO management, and post-ECMO events all contributing to CNS injury rates in these patients. Loss of cerebral autoregulation during severe arterial hypertension or hypotension, thromboembolic events, haemorrhage related to anticoagulation use, cerebral vasospasm, and secondary brain injury from reactive tissue oedema around an area of focal CNS injury have all been implicated in the genesis of brain injury in VA-ECMO patients. Although neurologic injury during VA-ECMO remains poorly defined in adult cohorts [16, 20–22], prior investigations comprehensively report neurologic complications occurrence in 6–17% in adults supported with VA-ECMO for postcardiotomy cardiorespiratory failure [20, 23, 24]. What seems even more illustrative, postmortem examination in adults supported with VA-ECMO has shown that neurologic injury may be clinically undetected in 23–50% of cases [25, 26]. In the current analysis, we saw neurologic complications more frequent in non-HTx centres. While this could not be accounted for in that type of analyses, the “over-delay” to ECMO commencement in the institutions with lower experience with circulatory support systems (be that ECMO or VADs) may have played a role in the excess of strokes in this population.

Finally, patients on ECMO bridged to HTx or mechanical circulatory support are reported only in the HTx units. Single patients in non-HTx institutions died while waiting for referral to HTx hub after the decision to transplant was met. From the technical standpoint, is noteworthy to underline that the were no differences in the ECMO duration between the two centres, yet notice must be made of statistical trend for the different ECMO location placement and weaning rate; that is, ECMO was instituted in the OR more frequently in non-HTx centres but again since delay to ECMO was seldom reported, we cannot address the issue whether this might had affected patient outcome. On the other hand, this may further suggest an easier applicability of ECMO in locations other than OR in HTx-centres with possibly prompt ECMO team availability as compared to the non-HTx units [27].

## Limitations

Current meta-analysis is based on observational, one-arm comparisons and because of that is more prone to confounding as compared to head-to-head comparisons. On the other hand, no RCT exist regarding analyzed topic and presumably will not be organized due to ethical issues. One limitation of the study may be underrepresentation of patients treated in non-HTx institutions. Random effect meta-analysis and inverse variance analysis was used to account for that fact. These methods appoint random weights also in a subgroup analysis which could overcome discrepancies between population sizes. There was no standard definition for secondary endpoints or risk of bias of the included studies. Conducting detailed subgroup analyses was precluded by not sufficient data on timing and location of implantation, type, status and duration of surgical procedure likewise other baseline characteristic.

## Conclusions

There was no apparent difference in survival after ECMO implantation for refractory PCS between centres which perform heart transplantations and those which do not. Potentially different risk profiles of patients in these centres must be taken account for before definite conclusions are drawn.

## Supplementary information

**Additional file 1.** Meta-analysis to Assess the Impact of Centre’s Heart Transplant Status and Volume on in-hospital Outcomes Following Extracorporeal Membrane Oxygenation for Refractory Post-cardiotomy Cardiogenic Shock.

## Data Availability

All data are available; corresponding author should be contacted to request the data.

## Abbreviations

95% CIs: 95% confidence intervals
AKI: Acute kidney injury
CNS: Central nervous system
CVE: Cerebrovascular event
CVVH: Continuous veno-venous hemofiltration
ECMO: Extracorporeal membrane oxygenation
ELSO: Extracorporeal life support organization
HTx: Heart transplantation
MCS: Mechanical circulatory support
OR: Odds ratio
PCS: Postcardiotomy cardiogenic shock
PRISMA: Preferred item reporting for systematic reviews and meta-analyses
ROBINS-I: Risk of Bias in not-randomized studies-of interventions
STEMI: ST segment elevation myocardial infarction
VAD: Ventricle assist device
VA-ECMO: Veno-arterial extracorporeal membrane oxygenation
