# Supplementary material for: The impact of Centre’s heart transplant status and volume on in-hospital outcomes following extracorporeal membrane oxygenation for refractory post-cardiotomy cardiogenic shock: a meta-analysis
Source: BMC Cardiovasc Disord. 2020 Jan 9;20:10. doi: 10.1186/s12872-019-01317-y (PMC6950870; doi:10.1186/s12872-019-01317-y)
Supplement: Supplementary file 1 — Additional file 1. Meta-analysis to Assess the Impact of Centre’s Heart Transplant Status and Volume on in-hospital Outcomes Following Extracorporeal Membrane Oxygenation for Refractory Post-cardiotomy Cardiogenic Shock. [file 12872_2019_1317_MOESM1_ESM.docx]

**Meta-analysis to Assess the Impact of Centre’s Heart Transplant Status and Volume on in-hospital Outcomes Following Extracorporeal Membrane Oxygenation for Refractory Post-cardiotomy Cardiogenic Shock**

- Impact of Transplant Status and Volume on ECMO results -

**SUPPLEMENTARY APPENDIX**

**References to included studies:**

1. Acheampong B, Johnson JN, Stulak JM, Dearani JA, Kushwaha SS, Daly RC, Haile DT, Schears GJ. Postcardiotomy ecmo support after high-risk operations in adult congenital heart disease. Congenit Heart Dis. 2016;11:751-755 1.
2. Bakhtiary F, Keller H, Dogan S, Dzemali O, Oezaslan F, Meininger D, Ackermann H, Zwissler B, Kleine P, Moritz A. Venoarterial extracorporeal membrane oxygenation for treatment of cardiogenic shock: Clinical experiences in 45 adult patients. J Thorac Cardiovasc Surg. 2008;135:382-388
3. Beckmann E, Ismail I, Cebotari S, Busse A, Martens A, Shrestha M, Kuhn C, Haverich A, Fegbeutel C. Right-sided heart failure and extracorporeal life support in patients undergoing pericardiectomy for constrictive pericarditis: A risk factor analysis for adverse outcome. Thorac Cardiovasc Surg. 2017;65:662-670
4. Beiras-Fernandez A, Deutsch MA, Kainzinger S, Kaczmarek I, Sodian R, Ueberfuhr P, Meiser B, Schmoeckel M, Reichart B, Brenner P. Extracorporeal membrane oxygenation in 108 patients with low cardiac output - a single-center experience. Int J Artif Organs. 2011;34:365-373
5. Biancari F, Dalen M, Perrotti A, Fiore A, Reichart D, Khodabandeh S, Gulbins H, Zipfel S, Al Shakaki M, Welp H, Vezzani A, Gherli T, Lommi J, Juvonen T, Svenarud P, Chocron S, Verhoye JP, Bounader K, Gatti G, Gabrielli M, Saccocci M, Kinnunen EM, Onorati F, Santarpino G, Alkhamees K, Ruggieri VG, Dell'Aquila AM. Venoarterial extracorporeal membrane oxygenation after coronary artery bypass grafting: Results of a multicenter study. Int J Cardiol. 2017;241:109-114
6. Burrell AJ, Pellegrino VA, Wolfe R, Wong WK, Cooper DJ, Kaye DM, Pilcher DV. Long-term survival of adults with cardiogenic shock after venoarterial extracorporeal membrane oxygenation. J Crit Care. 2015;30:949-956
7. Carroll BJ, Shah RV, Murthy V, McCullough SA, Reza N, Thomas SS, Song TH, Newton-Cheh CH, Camuso JM, MacGillivray T, Sundt TM, Semigran MJ, Lewis GD, Baker JN, Garcia JP. Clinical features and outcomes in adults with cardiogenic shock supported by extracorporeal membrane oxygenation. Am J Cardiol. 2015;116:1624-1630
8. Chen YC, Tsai FC, Chang CH, Lin CY, Jenq CC, Juan KC, Hsu HH, Chang MY, Tian YC, Hung CC, Fang JT, Yang CW. Prognosis of patients on extracorporeal membrane oxygenation: The impact of acute kidney injury on mortality. Ann Thorac Surg. 2011;91:137-142
9. Combes A, Leprince P, Luyt CE, Bonnet N, Trouillet JL, Leger P, Pavie A, Chastre J. Outcomes and long-term quality-of-life of patients supported by extracorporeal membrane oxygenation for refractory cardiogenic shock. Crit Care Med. 2008;36:1404-1411
10. Distelmaier K, Wiedemann D, Binder C, Haberl T, Zimpfer D, Heinz G, Koinig H, Felli A, Steinlechner B, Niessner A, Laufer G, Lang IM, Goliasch G. Duration of extracorporeal membrane oxygenation support and survival in cardiovascular surgery patients. J Thorac Cardiovasc Surg. 2017
11. Distelmaier K, Schrutka L, Binder C, Steinlechner B, Heinz G, Lang IM, Ristl R, Maurer G, Koinig H, Wiedemann D, Rutzler K, Niessner A, Goliasch G. Cardiac arrest does not affect survival in post-operative cardiovascular surgery patients undergoing extracorporeal membrane oxygenation. Resuscitation. 2016;104:24-27
12. Doll N, Fabricius A, Borger MA, Bucerius J, Doll S, Kramer K, Ullmann C, Schmitt DV, Walther T, Falk V, Mohr FW. Temporary extracorporeal membrane oxygenation in patients with refractory postoperative cardiogenic shock--a single center experience. J Card Surg. 2003;18:512-518
13. Elsharkawy HA, Li L, Esa WA, Sessler DI, Bashour CA. Outcome in patients who require venoarterial extracorporeal membrane oxygenation support after cardiac surgery. J Cardiothorac Vasc Anesth. 2010;24:946-951
14. Fiser SM, Tribble CG, Kaza AK, Long SM, Zacour RK, Kern JA, Kron IL. When to discontinue extracorporeal membrane oxygenation for postcardiotomy support. Ann Thorac Surg. 2001;71:210-214
15. Guihaire J, Dang Van S, Rouze S, Rosier S, Roisne A, Langanay T, Corbineau H, Verhoye JP, Flecher E. Clinical outcomes in patients after extracorporeal membrane oxygenation support for post-cardiotomy cardiogenic shock: A single-centre experience of 92 cases. Interact Cardiovasc Thorac Surg. 2017;25:363-369
16. Hsu PS, Chen JL, Hong GJ, Tsai YT, Lin CY, Lee CY, Chen YG, Tsai CS. Extracorporeal membrane oxygenation for refractory cardiogenic shock after cardiac surgery: Predictors of early mortality and outcome from 51 adult patients. Eur J Cardiothorac Surg. 2010;37:328-333
17. Kanji HD, Schulze CJ, Oreopoulos A, Lehr EJ, Wang W, MacArthur RM. Peripheral versus central cannulation for extracorporeal membrane oxygenation: A comparison of limb ischemia and transfusion requirements. Thorac Cardiovasc Surg. 2010;58:459-462
18. Ko WJ, Lin CY, Chen RJ, Wang SS, Lin FY, Chen YS. Extracorporeal membrane oxygenation support for adult postcardiotomy cardiogenic shock. Ann Thorac Surg. 2002;73:538-545
19. Lamarche Y, Chow B, Bedard A, Johal N, Kaan A, Humphries KH, Cheung A. Thromboembolic events in patients on extracorporeal membrane oxygenation without anticoagulation. Innovations (Phila). 2010;5:424-429
20. Li CL, Wang H, Jia M, Ma N, Meng X, Hou XT. The early dynamic behavior of lactate is linked to mortality in postcardiotomy patients with extracorporeal membrane oxygenation support: A retrospective observational study. J Thorac Cardiovasc Surg. 2015;149:1445-1450
21. Liden H, Wiklund L, Haraldsson A, Berglin E, Hultman J, Dellgren G. Temporary circulatory support with extra corporeal membrane oxygenation in adults with refractory cardiogenic shock. Scand Cardiovasc J. 2009;43:226-232
22. Liu KS, Tsai FC, Huang YK, Wu MY, Chang YS, Chu JJ, Lin PJ. Extracorporeal life support: A simple and effective weapon for postcardiotomy right ventricular failure. Artif Organs. 2009;33:504-508
23. Loforte A, Marinelli G, Musumeci F, Folesani G, Pilato E, Martin Suarez S, Montalto A, Lilla Della Monica P, Grigioni F, Frascaroli G, Menichetti A, Di Bartolomeo R, Arpesella G. Extracorporeal membrane oxygenation support in refractory cardiogenic shock: Treatment strategies and analysis of risk factors. Artif Organs. 2014;38:E129-141
24. Luo XJ, Wang W, Hu SS, Sun HS, Gao HW, Long C, Song YH, Xu JP. Extracorporeal membrane oxygenation for treatment of cardiac failure in adult patients. Interact Cardiovasc Thorac Surg. 2009;9:296-300
25. Mazzeffi MA, Sanchez PG, Herr D, Krause E, Evans CF, Rector R, McCormick B, Pham S, Taylor B, Griffith B, Kon ZN. Outcomes of extracorporeal cardiopulmonary resuscitation for refractory cardiac arrest in adult cardiac surgery patients. J Thorac Cardiovasc Surg. 2016;152:1133-1139
26. Meyer AL, Strueber M, Tomaszek S, Goerler A, Simon AR, Haverich A, Fischer S. Temporary cardiac support with a mini-circuit system consisting of a centrifugal pump and a membrane ventilator. Interact Cardiovasc Thorac Surg. 2009;9:780-783
27. Musial R, Ochonska K, Proc A, Stolinski J, Plicner D, Kapelak B, Drwila R. Veno-arterial extracorporeal membrane oxygenation as cardiogenic shock therapy support in adult patients after heart surgery. Kardiochir Torakochirurgia Pol. 2017;14:32-36
28. Papadopoulos N, Marinos S, El-Sayed Ahmad A, Keller H, Meybohm P, Zacharowski K, Moritz A, Zierer A. Risk factors associated with adverse outcome following extracorporeal life support: Analysis from 360 consecutive patients. Perfusion. 2015;30:284-290
29. Park SJ, Kim SP, Kim JB, Jung SH, Choo SJ, Chung CH, Lee JW. Blood lactate level during extracorporeal life support as a surrogate marker for survival. J Thorac Cardiovasc Surg. 2014;148:714-720
30. Peigh G, Cavarocchi N, Keith SW, Hirose H. Simple new risk score model for adult cardiac extracorporeal membrane oxygenation: Simple cardiac ecmo score. J Surg Res. 2015;198:273-279
31. Pokersnik JA, Buda T, Bashour CA, Gonzalez-Stawinski GV. Have changes in ecmo technology impacted outcomes in adult patients developing postcardiotomy cardiogenic shock? J Card Surg. 2012;27:246-252
32. Pontailler M, Demondion P, Lebreton G, Golmard JL, Leprince P. Experience with extracorporeal life support for cardiogenic shock in the older population more than 70 years of age. ASAIO J. 2017;63:279-284
33. Ranucci M, Ballotta A, Kandil H, Isgro G, Carlucci C, Baryshnikova E, Pistuddi V, Surgical, Clinical Outcome Research G. Bivalirudin-based versus conventional heparin anticoagulation for postcardiotomy extracorporeal membrane oxygenation. Crit Care. 2011;15:R275
34. Rastan AJ, Dege A, Mohr M, Doll N, Falk V, Walther T, Mohr FW. Early and late outcomes of 517 consecutive adult patients treated with extracorporeal membrane oxygenation for refractory postcardiotomy cardiogenic shock. J Thorac Cardiovasc Surg. 2010;139:302-311, 311 e301
35. Rousse N, Juthier F, Pincon C, Hysi I, Banfi C, Robin E, Fayad G, Jegou B, Prat A, Vincentelli A. Ecmo as a bridge to decision: Recovery, vad, or heart transplantation? Int J Cardiol. 2015;187:620-627
36. Rubino A, Costanzo D, Stanszus D, Valchanov K, Jenkins D, Sertic F, Fowles JA, Vuylsteke A. Central veno-arterial extracorporeal membrane oxygenation (c-va-ecmo) after cardiothoracic surgery: A single-center experience. J Cardiothorac Vasc Anesth. 2017
37. Russo CF, Cannata A, Lanfranconi M, Bruschi G, Milazzo F, Paino R, Martinelli L. Veno-arterial extracorporeal membrane oxygenation using levitronix centrifugal pump as bridge to decision for refractory cardiogenic shock. J Thorac Cardiovasc Surg. 2010;140:1416-1421
38. Saxena P, Neal J, Joyce LD, Greason KL, Schaff HV, Guru P, Shi WY, Burkhart H, Li Z, Oliver WC, Pike RB, Haile DT, Schears GJ. Extracorporeal membrane oxygenation support in postcardiotomy elderly patients: The mayo clinic experience. Ann Thorac Surg. 2015;99:2053-2060
39. Slottosch I, Liakopoulos O, Kuhn E, Deppe AC, Scherner M, Madershahian N, Choi YH, Wahlers T. Outcomes after peripheral extracorporeal membrane oxygenation therapy for postcardiotomy cardiogenic shock: A single-center experience. J Surg Res. 2013;181:e47-55
40. Slottosch I, Liakopoulos O, Kuhn E, Scherner M, Deppe AC, Sabashnikov A, Mader N, Choi YH, Wippermann J, Wahlers T. Lactate and lactate clearance as valuable tool to evaluate ecmo therapy in cardiogenic shock. J Crit Care. 2017;42:35-41
41. Truby L, Mundy L, Kalesan B, Kirtane A, Colombo PC, Takeda K, Fukuhara S, Naka Y, Takayama H. Contemporary outcomes of venoarterial extracorporeal membrane oxygenation for refractory cardiogenic shock at a large tertiary care center. ASAIO J. 2015;61:403-409
42. Tsai TY, Tsai FC, Fan PC, Chang CH, Lin CY, Chang WW, Lee SY, Hsu HH, Tian YC, Fang JT, Yang CW, Chen YC. Application of the age, creatinine, and left ventricular ejection fraction score for patients on extracorporeal membrane oxygenation. Artif Organs. 2017;41:146-152
43. Wang J, Han J, Jia Y, Zeng W, Shi J, Hou X, Meng X. Early and intermediate results of rescue extracorporeal membrane oxygenation in adult cardiogenic shock. Ann Thorac Surg. 2009;88:1897-1903
44. Wang JG, Han J, Jia YX, Zeng W, Hou XT, Meng X. Outcome of veno-arterial extracorporeal membrane oxygenation for patients undergoing valvular surgery. PLoS One. 2013;8:e63924
45. Wu MY, Lin PJ, Lee MY, Tsai FC, Chu JJ, Chang YS, Haung YK, Liu KS. Using extracorporeal life support to resuscitate adult postcardiotomy cardiogenic shock: Treatment strategies and predictors of short-term and midterm survival. Resuscitation. 2010;81:1111-1116
46. Xie HX, Yang F, Jiang CJ, Wang JH, Hou DB, Wang JG, Wang H, Hou XT. [predictors of in-hospital mortality in adult postcardiotomy cardiacgenic shock patients successfully weaned from venoarterial extracorporeal membrane oxygenation]. Zhonghua Yi Xue Za Zhi. 2017;97:929-933
47. Zhang R, Kofidis T, Kamiya H, Shrestha M, Tessmann R, Haverich A, Klima U. Creatine kinase isoenzyme mb relative index as predictor of mortality on extracorporeal membrane oxygenation support for postcardiotomy cardiogenic shock in adult patients. Eur J Cardiothorac Surg. 2006;30:617-620
48. Zhao Y, Xing J, Du Z, Liu F, Jia M, Hou X. Extracorporeal cardiopulmonary resuscitation for adult patients who underwent post-cardiac surgery. Eur J Med Res. 2015;20:83
49. Zhong Z, Jiang C, Yang F, Hao X, Xing J, Wang H, Hou X. Veno-arterial extracorporeal membrane oxygenation support in patients undergoing aortic surgery. Artif Organs. 2017;41:1113-1120
50. Ariyaratnam P, McLean LA, Cale AR, Loubani M. Extra-corporeal membrane oxygenation for the post-cardiotomy patient. Heart Fail Rev. 2014;19:717-725
51. Deschka H, Machner M, El Dsoki S, Alken A, Wimmer-Greinecker G. Central closed chest implantation of extracorporeal membrane oxygenation to prevent limb ischemia. Int J Artif Organs. 2013;36:687-692
52. Khorsandi M, Shaikhrezai K, Prasad S, Pessotto R, Walker W, Berg G, Zamvar V. Advanced mechanical circulatory support for post-cardiotomy cardiogenic shock: A 20-year outcome analysis in a non-transplant unit. J Cardiothorac Surg. 2016;11:29
53. Mikus E, Tripodi A, Calvi S, Giglio MD, Cavallucci A, Lamarra M. Centrimag venoarterial extracorporeal membrane oxygenation support as treatment for patients with refractory postcardiotomy cardiogenic shock. ASAIO J. 2013;59:18-23
54. Raffa GM, Gelsomino S, Sluijpers N, Meani P, Alenizy K, Natour E, Bidar E, Johnson DM, Makhoul M, Heuts S, Lozekoot P, Kats S, Schreurs R, Delnoij T, Montalti A, Sels JW, Poll MV, Roekaerts P, Maessen J, Lorusso R. In-hospital outcome of post-cardiotomy extracorporeal life support in adult patients: The 2007-2017 maastricht experience. Crit Care Resusc. 2017;19:53-61
55. Unosawa S, Sezai A, Hata M, Nakata K, Yoshitake I, Wakui S, Kimura H, Takahashi K, Hata H, Shiono M. Long-term outcomes of patients undergoing extracorporeal membrane oxygenation for refractory postcardiotomy cardiogenic shock. Surg Today. 2013;43:264-270

**Legend to Appendix Figures.**

**Appendix Figure 1**. PRISMA Flow-chart.

**Appendix Figure 2.** Funnel plot for publication bias analysis.

**Appendix Figure 3.** Meta-regression analysis of number of ECMOs per year on logit survival rate. HTx, heart transplantation; VAD, ventricle assist device; CIs, confidence intervals.

**Appendix Figure 4.** Meta-regression analysis of number of ECMOs per year on logit limb complications event rate. Abbreviations as in Appendix Figure 3.

**Appendix Figure 5.** Analysis of brain death following ECMO institution in HTx/VAD vs non-HTx/VAD centres. Squares represent point estimates of single studies; horizontal lines are respective 95% confidence intervals. Diamonds are indicative of subtotal and total pooled estimate.

**Appendix Figure 6.** Meta-regression analysis of number of ECMOs per year on logit neurologic complications event rate. Abbreviations as in Appendix Figure 3.

**Appendix Figure 7.** Meta-regression analysis of number of ECMOs per year on logit brain death event rate. Abbreviations as in Appendix Figure 3.

**Appendix Figure 8.** Meta-regression analysis of number of ECMOs per year on logit reoperations for bleeding event rate. Abbreviations as in Appendix Figure 3.

**Appendix Figure 9.** Meta-regression analysis of number of ECMOs per year on logit sepsis event rate. Abbreviations as in Appendix Figure 3.

**Appendix Figure 10.** Meta-regression analysis of number of ECMOs per year on logit acute kidney injury event rate. Abbreviations as in Appendix Figure 3.

**Appendix Figure 11.** Subgroup analysis to assess impact of centre volume on survival: arbitrary thresholds. Abbreviations as in Appendix Figure 3.

**Appendix Figure 12.** Subgroup analysis to assess impact of centre volume on survival: thresholds divided by mean and median number of ECMOs per year and divided in tertiles and quartiles. Abbreviations as in Appendix Figure 3.

**Appendix Figure 1.**

**
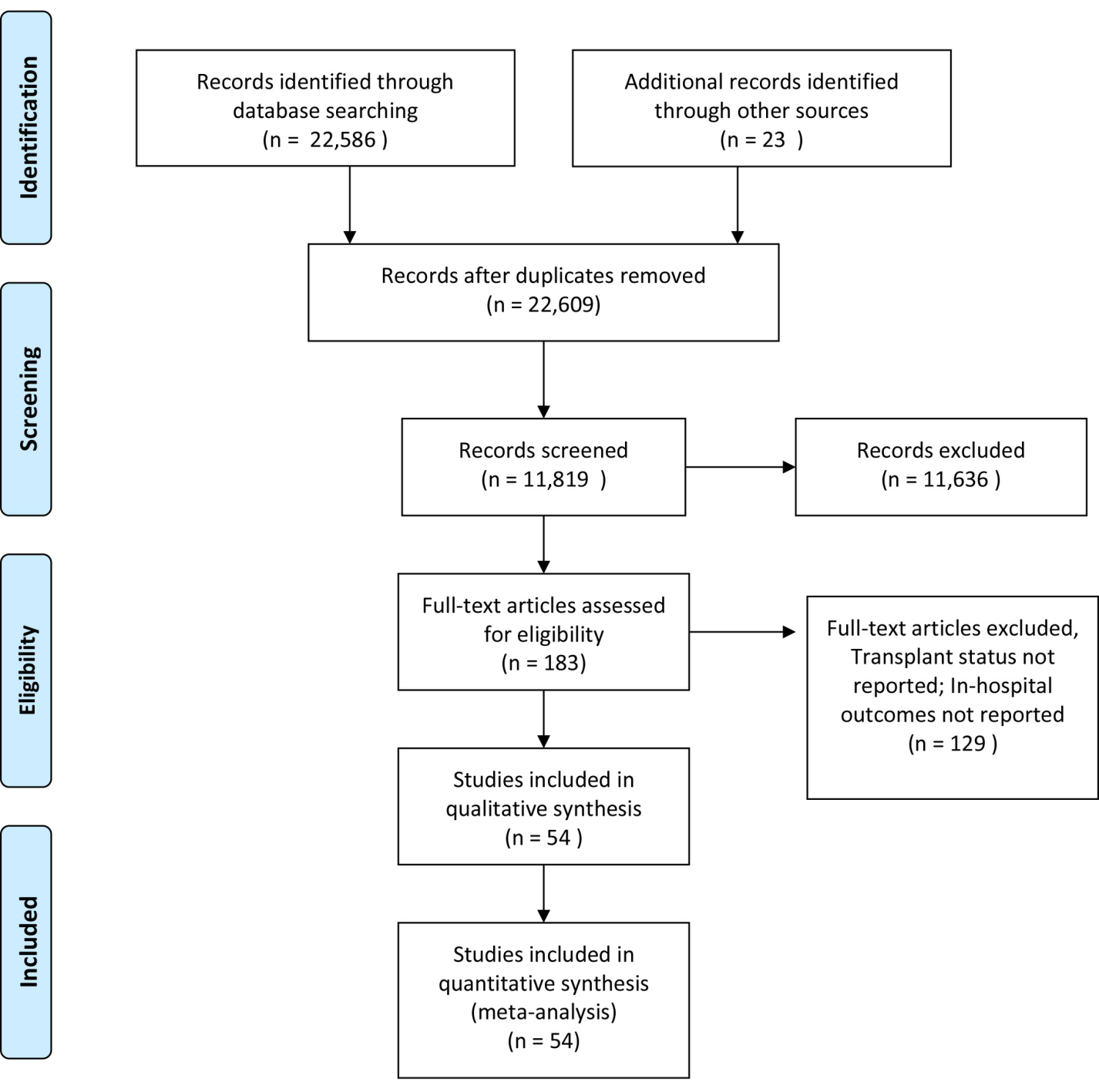
**

**Appendix Figure 2.**


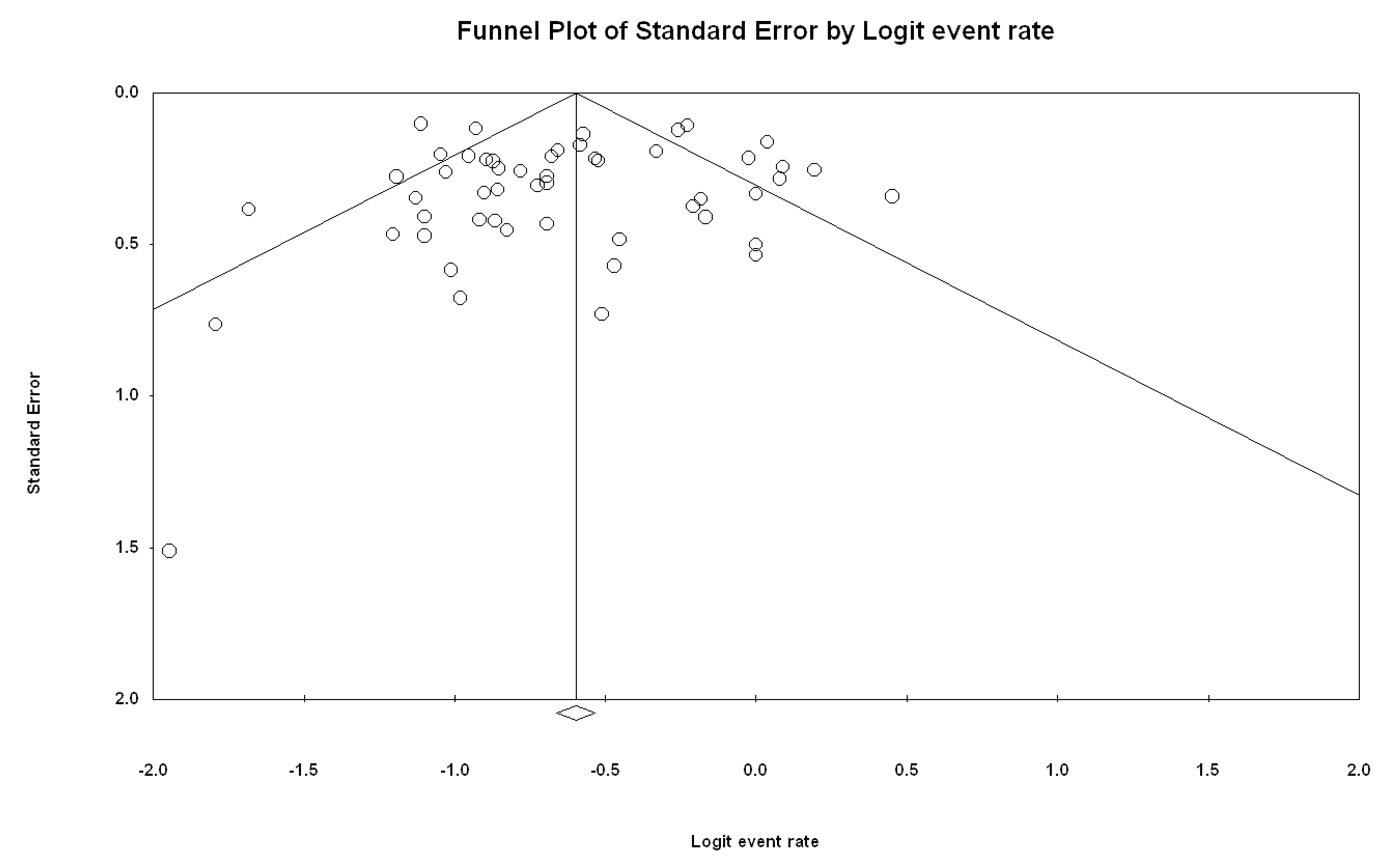


**Appendix Figure 3**.





**Appendix Figure 4**.



**Appendix Figure 5.**

**Appendix Figure 6.**

**

**

**Appendix Figure 7.**

**Appendix Figure 8.**

**Appendix Figure 9.**

**Appendix Figure 10.**

**Appendix Figure 11.**


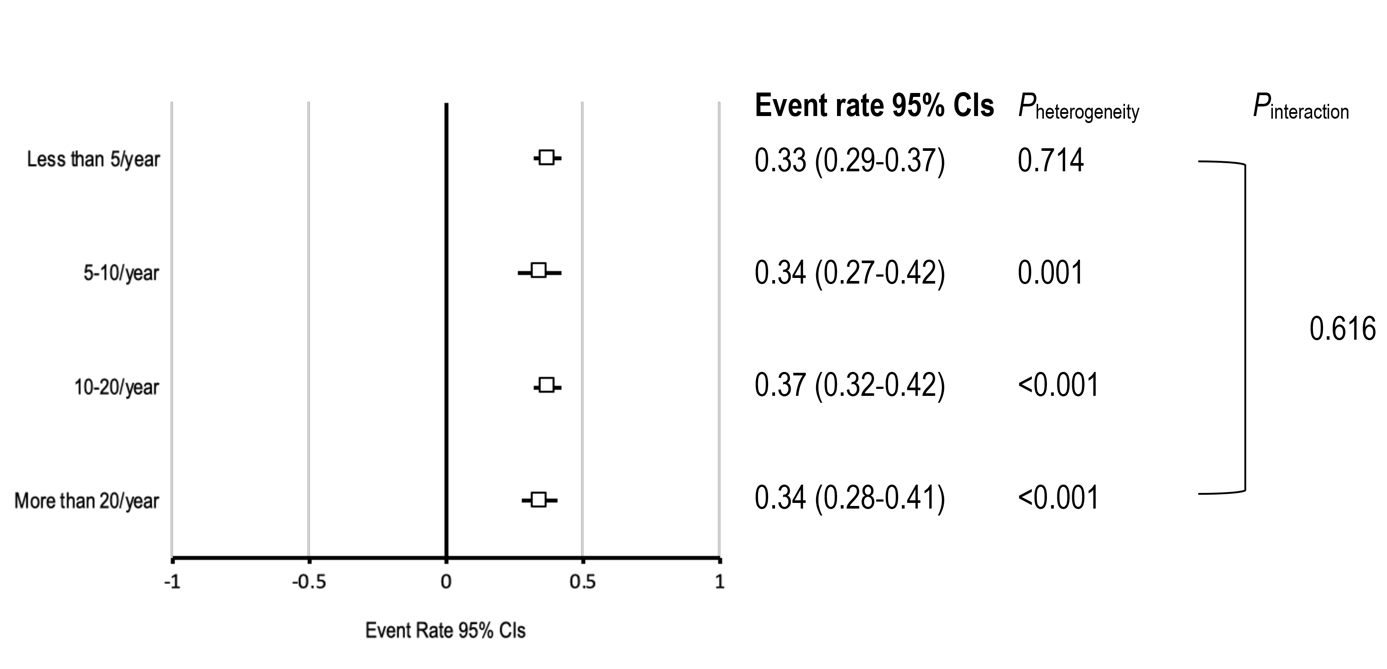


**Appendix Figure 12.**


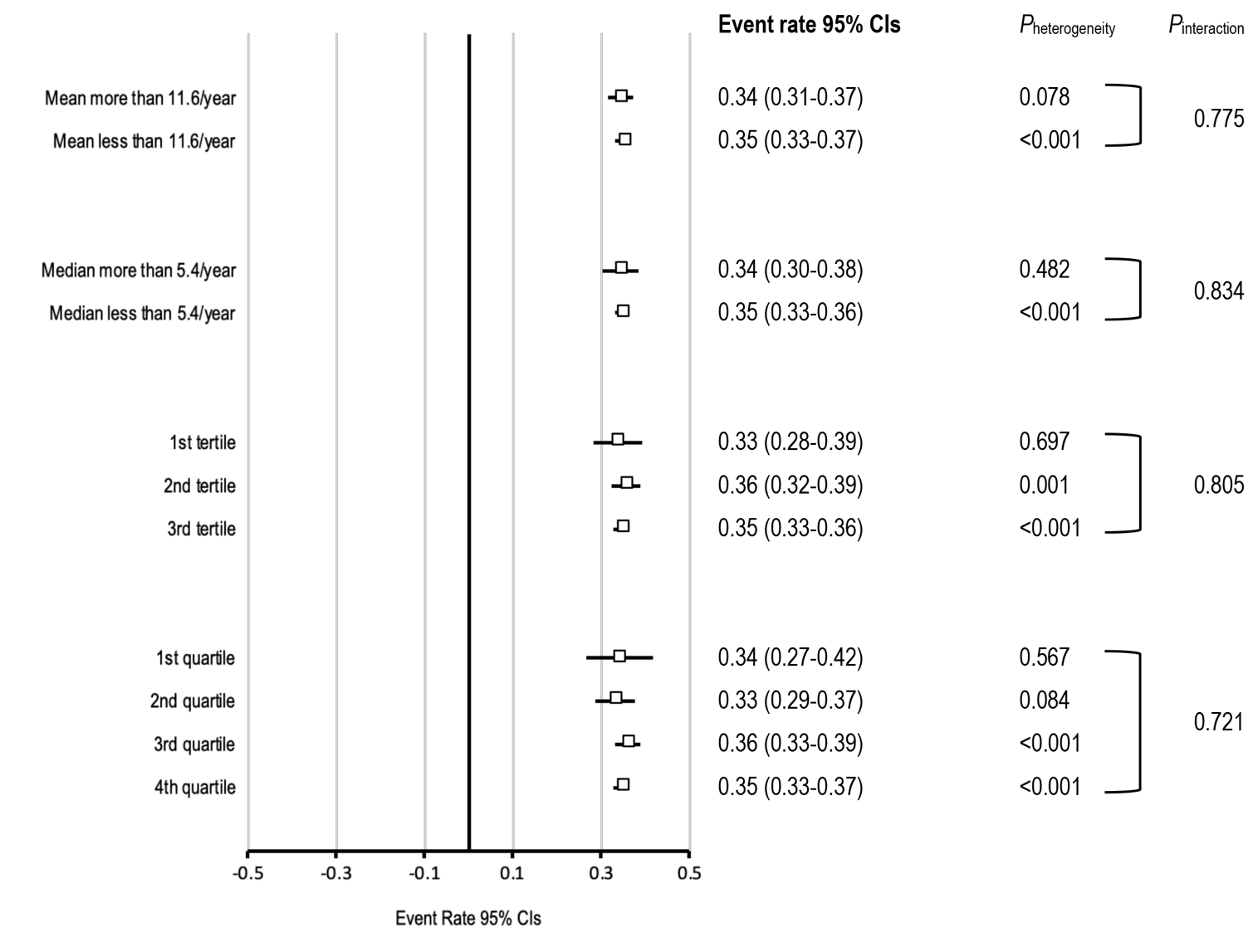


| **Appendix Table 1.** ROBINS-I tool bias assessment. | | | | | | | | | |
| --- | --- | --- | --- | --- | --- | --- | --- | --- | --- |
| Study | Bias due to confounding | Bias in selection of participants into the study | Bias in measurement of interventions | Bias due to departures from intended interventions | Bias due to missing data* | Bias in measurement of outcomes* | Bias in selection of reported result* | Overall bias | Cohen’s Kappa |
| Acheampong  2016 [19] | Critical | Critical | Serious | NA | Moderate | Moderate | Moderate | Moderate | 0.57 |
| Bakhtiary  2008 [20] | Critical | Low | Serious | NA | Moderate | Low | Low | Low | 0.71 |
| Beckmann  2017 [21] | Critical | Critical | Serious | NA | Low | Moderate | Moderate | Critical | 0.71 |
| Beiras-Fernandez  2011 [22] | Critical | Low | Low | NA | Low | Critical | Critical | Critical | 0.71 |
| Biancari  2017 [23] | Critical | Low | Serious | NA | Low | Low | Low | Low | 0.86 |
| Burrell  2015 [24] | Critical | Critical | Low | NA | Moderate | Moderate | Moderate | Moderate | 0.86 |
| Carroll  2015 [25] | Critical | Low | Moderate | NA | Serious | Critical | Serious | Critical | 0.57 |
| Chen  2011 [26] | Critical | Critical | Critical | NA | Serious | Critical | Critical | Critical | 0.86 |
| Combes  2008 [27] | Critical | Low | Low | NA | Serious | Critical | Critical | Critical | 0.86 |
| Distelmeier  2016 [28,29] | Critical | Low | Low | NA | Moderate | Critical | Serious | Critical | 0.86 |
| Doll  2003 [30] | Critical | Low | Low | NA | Moderate | Low | Low | Low | 0.71 |
| Elsharkawy  2010 [31] | Critical | Low | Low | NA | Low | Moderate | Moderate | Low | 0.71 |
| Fiser  2001 [32] | Critical | Low | Critical | NA | Moderate | Moderate | Moderate | Moderate | 0.86 |
| Guihaire  2017 [33] | Critical | Serious | Low | NA | Low | Moderate | Moderate | Moderate | 0.86 |
| Hsu  2009 [34] | Critical | Low | Low | NA | Moderate | Moderate | Moderate | Moderate | 0.86 |
| Kanji  2010 [35] | Critical | Low | Serious | NA | Serious | Moderate | Moderate | Serious | 1 |
| Ko  2002 [36] | Critical | Low | Critical | NA | Low | Moderate | Moderate | Critical | 0.86 |
| Lamarche  2010 [37] | Critical | Low | Moderate | NA | Moderate | Moderate | Moderate | Moderate | 0.57 |
| Li  2015 [38] | Critical | Low | Low | NA | Low | Moderate | Moderate | Low | 0.86 |
| Liden  2009 [39] | Critical | Moderate | Critical | NA | Moderate | Moderate | Moderate | Moderate | 0.57 |
| Liu  2009 [40] | Critical | Critical | Moderate | NA | Low | Moderate | Moderate | Moderate | 0.71 |
| Loforte  2014 [41] | Critical | Low | Critical | NA | Moderate | Moderate | Low | Critical | 0.71 |
| Luo  2009 [42] | Critical | Low | Low | NA | Moderate | Moderate | Low | Low | 1 |
| Mazeffi  2016 [43] | Critical | Low | Moderate | NA | Low | Moderate | Moderate | Moderate | 1 |
| Meyer  2009 [44] | Critical | Low | Low | NA | Moderate | Moderate | Moderate | Moderate | 0.86 |
| Musial  2017 [45] | Critical | Low | Critical | NA | Low | Serious | Moderate | Critical | 0.71 |
| Papadopoulos  2015 [46] | Critical | Low | Low | NA | Low | Moderate | Moderate | Low | 0.86 |
| Park  2014 [47] | Critical | Moderate | Low | NA | Low | Moderate | Moderate | Moderate | 0.86 |
| Peigh  2015 [48] | Critical | Low | Critical | NA | Serious | Critical | Critical | Critical | 0.57 |
| Pokersnik  2012 [49] | Critical | Serious | Critical | NA | Low | Moderate | Moderate | Critical | 0.71 |
| Pontailler  2017 [50] | Critical | Low | Serious | NA | Serious | Critical | Critical | Critical | 0.86 |
| Ranucci  2011 [51] | Critical | Critical | Critical | NA | Critical | Critical | Critical | Critical | 0.86 |
| Rastan  2010 [52] | Critical | Low | Low | NA | Low | Moderate | Moderate | Low | 0.71 |
| Rousse  2015 [53] | Critical | Low | Serious | NA | Moderate | Critical | Serious | Critical | 0.71 |
| Rubino  2017 [54] | Critical | Low | Critical | NA | Low | Moderate | Moderate | Critical | 0.71 |
| Russo  2010 [55] | Critical | Low | Low | NA | Low | Critical | Serious | Low | 0.71 |
| Saxena  2015 [9] | Critical | Low | Low | NA | Low | Moderate | Moderate | Low | 0.86 |
| Slottosch  2012 [56] | Critical | Low | Low | NA | Low | Low | Moderate | Low | 0.86 |
| Slottosch  2017 [57] | Critical | Low | Low | NA | Moderate | Moderate | Moderate | Moderate | 0.86 |
| Truby  2015 [58] | Critical | Low | Serious | NA | Moderate | Critical | Serious | Critical | 0.86 |
| Tsai  2016 [59] | Critical | Critical | Critical | NA | Serious | Critical | Critical | Critical | 0.57 |
| Wang  2009 [60] | Critical | Low | Low | NA | Moderate | Low | Moderate | Low | 0.71 |
| Wang  2013 [61] | Critical | Critical | Low | NA | Low | Low | Moderate | Low | 0.86 |
| Wu  2010 [62] | Critical | Low | Critical | NA | Low | Low | Low | Low | 1 |
| Xie  2017 [63] | Critical | Low | Critical | NA | Critical | Critical | Critical | Critical | 0.86 |
| Zhang  2006 [64] | Critical | Low | Critical | NA | Low | Serious | Moderate | Critical | 0.86 |
| Zhao  2015 [65] | Critical | Critical | Critical | NA | Low | Moderate | Moderate |  | 0.71 |
| Zhong  2017 [66] | Critical | Critical | Low | NA | Low | Serious | Moderate | Critical | 0.57 |
| Ariyaratnam  2014 [67] | Critical | Low | Critical | NA | Low | Critical | Serious | Critical | 1 |
| Deschka  2013 [68] | Critical | Low | Serious | NA | Moderate | Low | Low | Low | 0.86 |
| Khorsandi  2016 [69] | Critical | Low | Serious | NA | Low | Low | Low | Low | 0.86 |
| Mikus  2013 [70] | Critical | Low | Low | NA | Low | Moderate | Moderate | Low | 0.71 |
| Raffa  2017 [71] | Critical | Low | Serious | NA | Low | Moderate | Low | Low | 0.71 |
| Unosawa  2012 [72] | Critical | Low | Low | NA | Low | Moderate | Moderate | Low | 0.86 |
| *When multiple outcomes were reported for a study, the highest level of bias at the outcome level is reported in the table. | | | | | | | | |  |

| Appendix Table 2. Procedural characteristics | | | | | | | | | | | | | | | | | | | | |
| --- | --- | --- | --- | --- | --- | --- | --- | --- | --- | --- | --- | --- | --- | --- | --- | --- | --- | --- | --- | --- |
| Study | | Post-op IABP (n) | Peripheral cannulation (n) | | ECMO at index procedure (n) | | ECMO at OR/ICU | | Distal perfusion (n) | | LV venting (n) | | ECMO | | | | | | | |
|  |  |  |  |  |  |  |  |  |  |  |  |  | Duration | | weaning (%) | | Bridge to VAD (n) | | Bridge to HTx (n) | |
| HTx/VAD centres | | | | | | | | | | | | | | | | | | | | |
|  | Acheampong 2016 | 14 | | NR | | NR | | 20/4 | | NR | | NR | | 8.4 (0.8–35.4) days | | 75.0 | | 1 | | 0 |
|  | Bakhtiary 2008 | 30 | | 37 | | NR | | 30/15 | | NR | | NR | | 6.4 ± 4.5 days | | 55.6 | | 5 | | 2 |
|  | Beckmann 2017 | NR | | 8 | | NR | | NR | | 8 | | NR | | 12.0 ± 5.9 days | | 87.5 | | 1 | | 0 |
|  | Beiras-Fernandez 2011 | 36 | | NR | | NR | | NR | | NR | | NR | | 4.4 ± 4.0 days | | NR | | NR | | NR |
|  | Biancari 2017 | 47 | | 89 | | 76 | | NR | | 66 | | 5 | | 6.4 ± 5.6 days | | 48.6 | | 6 | | 0 |
|  | Burrell 2015 | NR | | NR | | NR | | NR | | NR | | NR | | 7 (IQR: 5-8) days | | 100 | | NR | | NR |
|  | Carroll 2015 | 13 | | 92 | | NR | | 61/31** | | NR | | NR | | 94 (IQR: 43-172) hours | | 69 | | 29 | | 2 |
|  | Chen 2011* | NR | | NR | | NR | | NR | | NR | | NR | | 133 ± 12 hours | | 54.9 | | NR | | NR |
|  | Combes 2008 | NR | | NR | | NR | | NR | | NR | | NR | | NR | | NR | | NR | | NR |
|  | Distelmeier 2016/2017 | 44 | | 347 | | NR | | NR | | NR | | NR | | 4 (IQR: 2–7) days | | NR | | 4 | | 3 |
|  | Doll 2003 | 95 | | 26 | | NR | | 70/25 | | NR | | NR | | 2.8 ± 2.1 days | | 47.4 | | 8 | | 3^†^ |
|  | Elsharkawy 2010 | 22 | | 77 | | NR | | NR | | NR | | NR | | NR | | NR | | 28 | | 25 |
|  | Fiser 2001 | NR | | NR | | NR | | 22/29 | | NR | | NR | | 67.5 hours | | 31.4 | | NR | | NR |
|  | Guihaire 2017 | 25 | | 78 | | 43 | | 80/12 | | NR | | 13 | | 6 days | | 48 | | 2 | | 2 |
|  | Hsu 2009 | 51 | | 51 | | NR | | NR | | NR | | NR | | 7.5 ± 6.7 days | | 52.9 | | 0 | | 3 |
|  | Kanji 2010 | 36 | | 22 | | NR | | NR | | NR | | NR | | 2.5 (IQR: 1.0-4.8) days | | NR | | NR | | 23 |
|  | Ko 2002 | 54 | | 61 | | 39 | | 23/30 | | 20 | | NR | | NR | | 60.5 | | 2 | | 2 |
|  | Lamarche 2010 | NR | | 8 | | NR | | 16/8 | | NR | | NR | | 48 ± 65.5 hours | | 58.3 | | 3 | | 3 |
|  | Li 2015 | 73 | | 123 | | 61 | | NR | | 123 | | NR | | 4.3 days | | 56.1 | | NR | | NR |
|  | Liden 2009 | 14 | | NR | | NR | | NR | | NR | | NR | | 5.5 ± 4.9 days | | NR | | 2 | | 2 |
|  | Liu 2009 | 10 | | 14 | | 9 | | NR | | NR | | NR | | 71 ± 52 hours | | 64.3 | | NR | | NR |
|  | Loforte 2014 | 155 | | 79 | | NR | | NR | | NR | | NR | | 9.56 (2–43) days | | 56.8 | | 1 | | 4 |
|  | Luo 2009 | 11 | | NR | | NR | | 15/30 | | NR | | 0 | | 131.6 hours | | 66.7 | | NR | | 2 |
|  | Mazeffi 2016 | 3 | | 9 | | 3 | | NR | | NR | | 0 | | 3 (IQR: 1-8) days | | 100 | | 2 | | NR |
|  | Meyer 2009 | NR | | 18 | | NR | | NR | | 18 | | NR | | 4 ± 4 days | | 66.7 | | NR | | NR |
|  | Musial 2017 | NR | | 27 | | 27 | | NR | | NR | | NR | | 8.89 days | | NR | | NR | | NR |
|  | Papadopoulos 2015 | NR | | 324 | | NR | | NR | | NR | | NR | | 7 ± 1 days | | 58.1 | | 6 | | 2 |
|  | Park 2014 | NR | | 115 | | 41 | | NR | | 115 | | NR | | 71.3 (0.5-713) hours | | 40.8 | | NR | | 2 |
|  | Peigh 2015 | NR | | NR | | NR | | NR | | NR | | NR | | 9.2 ± 6.1 days | | NR | | NR | | NR |
|  | Pokersnik 2012 | 29 | | 32 | | NR | | NR | | 32 | | NR | | 3.8 ± 3.4 days | | 55.1 | | 2 | | 0 |
|  | Pontailler 2017 | NR | | NR | | NR | | NR | | NR | | NR | | 5.6 ± 4.3 days* | | 41.1* | | NR | | NR |
|  | Ranucci 2011 | NR | | 0 | | 9/2 | | NR | | NR | | NR | | 122.1 ± 80.5 hours | | 63.6 | | NR | | NR |
|  | Rastan 2010 | 383 | | 203 | | NR | | NR | | 121 | | NR | | 3.28 ± 2.85 days | | 63.3 | | 15 | | 5 |
|  | Rousse 2015 | 5 | | 25 | | NR | | NR | | 29 | | 29 | | 8 (1-86) days | | 50.0 | | 7 | | 13 |
|  | Rubino 2017 | NR | | NR | | 63 | | NR | | NR | | NR | | 5 (IQR: 3-8) days | | 57.4 | | 3 | | 3 |
|  | Russo 2010 | 3 | | 1 | | NR | | NR | | NR | | NR | | 7.0 ± 5.2 days | | 66.7 | | 0 | | 1 |
|  | Saxena 2015^†^ | NR | | 15 | | NR | | NR | | NR | | NR | | 103.8 ± 74.3 hours | | 53.3 | | NR | | NR |
|  | Slottosch 2012 | 72 | | 77 | | 34 | | 34/43 | | 77 | | NR | | 79 ± 57 hours | | 62.3 | | NR | | NR |
|  | Slottosch 2017 | 83 | | 72 | | 40 | | NR | | NR | | NR | | 117 ± 78 hours | | 43.2* | | NR | | NR |
|  | Truby 2015 | NR | | 45 | | NR | | 71/108 | | 9 | | NR | | 3.58 (IQR: 1.6 –5.9) days | | NR | | 52 | | NR |
|  | Tsai 2016 | NR | | NR | | NR | | NR | | NR | | NR | | 7 ± 5 days* | | 70.5* | | NR | | NR |
|  | Wang 2009 | 19 | | NR | | NR | | 36/26 | | NR | | 0 | | 61 ± 37 hours | | 64.5 | | NR | | NR |
|  | Wang 2013 | 41 | | NR | | 37 | | 37/50 | | 37 | | NR | | 61 ± 37 hours | | 58.6 | | NR | | NR |
|  | Wu 2010 | NR | | NR | | NR | | 98/12 | | NR | | NR | | 143 ± 112 hours | | 60.9 | | 2 | | 0 |
|  | Xie 2017*** | NR | | NR | | NR | | NR | | NR | | NR | | NR | | 64.8 | | NR | | NR |
|  | Zhang 2006 | 10 | | 19 | | NR | | NR | | NR | | NR | | 2.7 ± 1.7 days | | 43.8 | | NR | | NR |
|  | Zhao 2015 | 16 | | 23 | | 9 | | NR | | NR | | NR | | 115.23 ± 70.17 hours | | 66.7 | | 1** | | NR |
|  | Zhong 2017 | 9 | | 29 | | 24 | | 24/12 | | NR | | NR | | 77.5 ± 34.5 hours | | 66.7 | | NR | | NR |
| Non-HTx/VAD centres | | | | | | | | | | | | | | | | | | | | |
|  | Ariyaratnam 2014 | NR | | 0 | | NR | | NR | | NR | | NR | | 5.6 ± 3.4 days | | 50.0 | | NR | | NR |
|  | Deschka 2013 | 25 | | 0*** | | NR | | NR | | NR | | NR | | 8.7 ± 3.9 days | | 42.9 | | NR | | NR |
|  | Khorsandi 2016 | NR | | 5 | | NR | | NR | | NR | | NR | | 5.5 ± 8.9 days | | NR | | NR | | NR |
|  | Mikus 2013 | 13 | | 6 | | 12 | | NR | | 14 | | 14 | | 5 days | | 50 | | 0 | | 0 |
|  | Raffa 2017 | 23 | | 30 | | 48 | | 48/38 | | NR | | NR | | 5 days | | 49 | | NR | | NR |
|  | Unosawa 2012 | 39 | | 32 | | 33 | | 33/14 | | NR | | NR | | 63.5 ± 61.5 hours | | 61.7 | | 0 | | 0 |

* Reported for entire study population including non-PCS patients

** 24 (23%) in the cardiac catheterization laboratory, 16 (16%) at the bedside, and 1 (1%) in the emergency department

*** Central cannulation cohort alone; peripheral venous cannulation in 28 patients

^†^VAD and subsequent heart transplantation

Values are reported as mean ± SD, unless reported otherwise in the original manuscript. HTx, heart transplantation; VAD, ventricular assist device; IQR, interquartile range; NR, not reported.

Numbers of patients in single studies do not always match the total n. of patients in the original manuscripts since only PCS subgroups were considered.

| Appendix Table 3. Reported causes of in-hospital/30-day death | | |  |
| --- | --- | --- | --- |
| Study | | **On ECMO only** | **After weaning/in-hospital/30-day death** |
| HTx/VAD centres | | |  |
|  | Acheampong 2016 | NR | 1 sepsis, 1 massive bleeding, 2 pulmonary; remaining: multiorgan failure or cardiac related |
|  | Bakhtiary 2008 | 18 persistent heart failure | 12 multiorgan failure |
|  | Beckmann 2017 | 1 sepsis | 1 biventricular failure, 1 low cardiac output, 1 cerebral haemorrhage, 1 multiorgan failure |
|  | Beiras-Fernandez 2011 | refractory ventricular failure 40.6%, bleeding 23.2%, isolated persistent low cardiac output 21.7%, thromboembolism 8.7%, allograft failure 5.8% | NR |
|  | Biancari 2017 | NR | NR |
|  | Burrell 2015 | NR | NR |
|  | Carroll 2015 | NR | multiorgan failure in 52 patients, neurologic injury in 9 patients, bleeding in 7 patients, hypoxemic respiratory failure in 2 patients, and other etiologies in the remaining 5 patients |
|  | Chen 2011* | NR | NR |
|  | Combes 2008 | mainly due to refractory multiorgan failure with 14 within 24 hrs of onset | NR |
|  | Distelmeier 2016/2017 | NR | NR |
|  | Doll 2003 | NR | refractory myocardial failure in 71%, multisystem organ failure in 13%, sepsis in 7%, and other causes in 9% |
|  | Elsharkawy 2010 | NR | NR |
|  | Fiser 2001 | 27 cardiac failures, 12 strokes, 3 multisystem organ failures, 1 respiratory insufficiency | NR |
|  | Guihaire 2017 | NR | NR |
|  | Hsu 2009 | NR | main causes were pulmonary infections |
|  | Kanji 2010 | NR | NR |
|  | Ko 2002 | 16 multiple organ failure, 5 uncontrolled bleedings, 3 brain deaths, 2 circulatory shocks, 1 refractory ventricular arrhythmia, 1 myocardial infarction, 1 acute rejection, 1 graft failure | 17 multiple organ failure, 3 persistent heart failure, 1 ventricular fibrillation |
|  | Lamarche 2010 | NR | NR |
|  | Li 2015 | NR | NR |
|  | Liden 2009 | 8 multiorgan failure, 6 poor cardiac function, 1 brain damage, 1 retroperitoneal bleeding, 1 fungal sepsis, 1 vasoplegia, 1 unknown | NR |
|  | Liu 2009 | NR | 4 haemodynamic unstable, 1 multiorgan failure, 1 sepsis, 1 other |
|  | Loforte 2014 | mostly associated with sepsis and brain death | NR |
|  | Luo 2009 | 6 persistent heart failure without improvement, 4 multiorgan failure and others | 5 sepsis and other |
|  | Mazeffi 2016 | NR | 7 multiple organ failure, 4 brain injury, 3 sepsis, 1 hemorrhage, 1 repeat arrest |
|  | Meyer 2009 | NR | 5 multiorgan failure, 4 non-recoverable myocardial failure, 3 sepsis, 1 intracerebral bleeding |
|  | Musial 2017 | NR | NR |
|  | Papadopoulos 2015 | NR | the main cause of death was sepsis (69%) |
|  | Park 2014 | NR | 6 multiorgan failure, 3 profound cardiac failure, 2 sepsis, 2 pan-peritonitis, 2 aortic ruptures, 1 intracerebral haemorrhage, 1 gastrointestinal bleeding |
|  | Peigh 2015 | 8 anoxic brain injuries, 8 strokes, 3 irreversible cardiac dysfunctions, 3 sepsis, 2 irreversible lung diseases, 1 abdominal compartment syndrome with hepatorenal syndrome, 1 failure to control bleeding | 5 sepsis, 4 neurologic injuries, 2 acute myocardial infraction because of stent thrombosis, 1 family’s withdrawal because of failure to thrive, and one case of each of the following: pulseless electric activity after internal defibrillator placement, persistent loss of cardiac activity despite biventricular assist device placement, acute failure of left ventricular assist device placement with persistent low flow and malperfusion, nonresectable cardiac metastasis (adenocarcinoma), and severe coagulopathy and multiple bleeding |
|  | Pokersnik 2012 | NR | NR |
|  | Pontailler 2017 | multiorgan failure for all patients | NR |
|  | Ranucci 2011 | NR | NR |
|  | Rastan 2010 | NR | the main reason for death was cardiac |
|  | Rousse 2015 | 21 multiple organ failures, 12 intra-abdominal bleeding, 4 strokes, 3 non-beating hearts, 7 haemorrhage or sepsis, 2 mesenteric ischemias (for entire study population of 124 patients) | NR |
|  | Rubino 2017 | NR | causes of death were mostly related to cardiac disease |
|  | Russo 2010 | NR | 6 multiorgan failures, 1 pulmonary artery thrombosis, 1 thoracic bleeding |
|  | Saxena 2015^†^ | NR | 30 cardiac causes, 13 multiorgan failures, 6 septicemias |
|  | Slottosch 2012 | NR | cardiac causes (50.9%), sepsis (29.1%), cerebrovascular complications (1.8%), bleeding (10.9%), gastrointestinal complications (7.3%) |
|  | Slottosch 2017 | NR | 60 cardiac deaths, 22 sepsis, 6 mesenterial ischemias, 5 cerebral deaths, 5 untreatable bleedings and 4 others (for entire cohort of 139 patients) |
|  | Truby 2015 | NR | NR |
|  | Tsai 2016 | NR | NR |
|  | Wang 2009 | 11 persistent heart failures, 7 sepsis with consecutive multiorgan failure, 2 cerebral infarction and bleeding, 2 disseminated intravascular coagulation | main cause was sepsis with consecutive multiorgan failure |
|  | Wang 2013 | 22 persistent heart failure without any improvement in cardiac function, 10 sepsis, 3 disseminated intravascular coagulation, 1 cerebral infarction and bleeding | sepsis with consecutive multiorgan failure for all weaned |
|  | Wu 2010 | NR | 8 excessive mediastinal bleeding, 15 profound cardiac failure, 6 severe brain damage, 3 bowel infarction, 2 massive air embolism, 28 sepsis with multiple organ failure |
|  | Xie 2017*** | NR | NR |
|  | Zhang 2006 | NR | NR |
|  | Zhao 2015 | NR | the main cause of mortality was multiple system organ failure (50.0 %) |
|  | Zhong 2017 | NR | 7 sepsis with subsequent multi-organ failure, 6 persistent heart failures, 3 disseminated intravascular coagulation, 2 cerebral infarction |
| Non-HTx/VAD centres | | |  |
|  | Ariyaratnam 2014 | NR | NR |
|  | Deschka 2013 | NR | NR |
|  | Khorsandi 2016 | NR | 6 biventricular failures, 2 septic shocks, 1 major cardiovascular incident, 1 major haemorrhage, 1 respiratory failure, 1 multiorgan failure |
|  | Mikus 2013 | multiple organ failure for all | NR |
|  | Raffa 2017 | NR | the main reason for death was a cardiac event, followed by multiorgan failure and neurological complications |
|  | Unosawa 2012 | 7 intractable heart failure, 5 multiorgan failure, 4 brain death, 2 severe coagulopathic bleeding | 8 multiorgan failures, 2 heart failures, 2 cardiac ruptures, 2 brain deaths, 1 pneumonia |

NR, not reported

| Appendix Table 4. AKI definitions | | |
| --- | --- | --- |
| Study | | AKI definition |
|  |  |  |
| HTx/VAD centres | | |
|  | Acheampong 2016 | Renal insufficiency requiring hemodialysis |
|  | Bakhtiary 2008 | Renal failure requiring continuous venovenous hemofiltration |
|  | Beckmann 2017 | N/A |
|  | Beiras-Fernandez 2011 | N/A |
|  | Biancari 2017 | Renal replacement therapy |
|  | Burrell 2015 | N/A |
|  | Carroll 2015 | N/A |
|  | Chen 2011* | N/A |
|  | Combes 2008 | N/A |
|  | Distelmeier 2016/2017 | N/A |
|  | Doll 2003 | Renal failure requiring hemofiltration |
|  | Elsharkawy 2010 | Acute renal failure requiring dialysis |
|  | Fiser 2001 | Renal failure requiring dialysis |
|  | Guihaire 2017 | N/A |
|  | Hsu 2009 | Renal failure requiring continuous venovenous hemofiltration |
|  | Kanji 2010 | N/A |
|  | Ko 2002 | Acute renal failure requiring dialysis |
|  | Lamarche 2010 | N/A |
|  | Li 2015 | Postoperative continuous renal replacement therapy |
|  | Liden 2009 | Postoperative dialysis |
|  | Liu 2009 | Acute renal failure requiring hemodialysis |
|  | Loforte 2014 | Continuous veno-venous hemofiltration on ECMO |
|  | Luo 2009 | Acute renal failure treated by continuous renal replacement therapy |
|  | Mazeffi 2016 | Acute renal failure requiring renal replacement therapy |
|  | Meyer 2009 | Renal failure requiring hemodialysis |
|  | Musial 2017 | Acute renal failure requiring continuous renal replacement therapy |
|  | Papadopoulos 2015 | Acute renal failure requiring continuous venovenous hemofiltration |
|  | Park 2014 | N/A |
|  | Peigh 2015 | N/A |
|  | Pokersnik 2012 | An increase in serum creatinine to >2.0, and two times the most recent preoperative creatinine level or a new requirement for dialysis postoperatively |
|  | Pontailler 2017 | N/A |
|  | Ranucci 2011 | N/A |
|  | Rastan 2010 | N/A |
|  | Rousse 2015 | N/A |
|  | Rubino 2017 | Renal impairment requiring continuous veno-venous hemofiltration |
|  | Russo 2010 | N/A |
|  | Saxena 2015^†^ | N/A |
|  | Slottosch 2012 | Oliguria (less than 0.5 mL/kg/h) and a doubling of postoperative creatinine values with the need for hemodialysis |
|  | Slottosch 2017 | Oliguria (<0.5 ml/kg/h) and a doubling of postoperative creatinine values with need for hemodialysis |
|  | Truby 2015 | N/A |
|  | Tsai 2016 | N/A |
|  | Wang 2009 | Renal failure requiring continuous venovenous hemofiltration |
|  | Wang 2013 | PCS-related renal failure requiring continuous venovenous hemofiltration (e.g. renal replacement therapy) |
|  | Wu 2010 | Renal failure requiring continuous arteriovenous hemofiltration |
|  | Xie 2017*** | N/A |
|  | Zhang 2006 | N/A |
|  | Zhao 2015 | Hemodialysis on ECMO |
|  | Zhong 2017 | N/A |
| Non-HTx/VAD centres | | |
|  | Ariyaratnam 2014 | N/A |
|  | Deschka 2013 | Acute renal failure requiring continuous veno-venous hemodiafiltration |
|  | Khorsandi 2016 | Renal failure requiring renal replacement therapy |
|  | Mikus 2013 | Renal failure requiring continuous venovenous hemofiltration |
|  | Raffa 2017 | N/A |
|  | Unosawa 2012 | N/A |

N/A, not available, ECMO, extracorporeal membrane oxygenation; PCS, postcardiotomy shock.
